# Models of protein production along the cell cycle: An investigation of possible sources of noise

**DOI:** 10.1371/journal.pone.0226016

**Published:** 2020-01-16

**Authors:** Renaud Dessalles, Vincent Fromion, Philippe Robert

**Affiliations:** 1 Dept. of Biomathematics, UCLA, Los Angeles, CA, United States of America; 2 MaIAGE, INRA, Université Paris-Saclay, Jouy-en-Josas, France; 3 INRIA de Paris, Paris, France; Universitat Pompeu Fabra, SPAIN

## Abstract

In this article, we quantitatively study, through stochastic models, the effects of several intracellular phenomena, such as cell volume growth, cell division, gene replication as well as fluctuations of available RNA polymerases and ribosomes. These phenomena are indeed rarely considered in classic models of protein production and no relative quantitative comparison among them has been performed. The parameters for a large and representative class of proteins are determined using experimental measures. The main important and surprising conclusion of our study is to show that despite the significant fluctuations of free RNA polymerases and free ribosomes, they bring little variability to protein production contrary to what has been previously proposed in the literature. After verifying the robustness of this quite counter-intuitive result, we discuss its possible origin from a theoretical view, and interpret it as the result of a mean-field effect.

## Introduction

For some time now, fluorescent microscopy methods have provided quantitative measurements of gene expression on the level of individual cells, see for instance [1, 2]. Measurements have shown that protein production is a highly variable process, even for genetically identical cells in constant environmental conditions. The fluctuations can negatively affect genetic expression and impact the behavior of the cell (see [3]), or, on the contrary, beneficially participate in strategies to adapt to a changing environment [4, 5].

More recently, [6] performed an extensive quantification of the variability of the gene expression of around a thousand different genes in *E. coli*. One experiment per gene was performed in their study: for each cell of a population, the total fluorescence of the protein associated with that gene was measured; this quantity was then normalized by cell volume and the fluorescence of a single protein (see the Supplementary Material of [6]), resulting in the protein concentration in each cell of the population. Furthermore, for a significant number of the genes considered, messenger RNA production is also quantified in each cell: using an mRNA-sequencing technique (RNA-seq), they were able to estimate the average production of 841 different types of mRNA, and using fluorescence *in situ* hybridization, they could even measure some types of mRNA (137 types) with single molecule precision in each cell of a population. Statistics over the population then gave the average concentration of each protein and RNA messenger, as well as the coefficient of variation (CV) for both quantities.

By comparing the behavior of the CV with that associated with the classic two-stage model of gene expression proposed in [7, 8] and reviewed in [9] (see below), Taniguchi et al. show that the behavior of the messengers’ variability with respect to their abundances resembles the one expected on the basis of the two-stage model. For protein variability, they identify two regimes of protein variability depending on the average protein concentration. For infrequently expressed proteins, the protein CV is shown to be inversely proportional to the average concentration, in accordance with the one expected with the two-stage model (as it is shown in [6]). By contrast, for highly produced proteins, the CV becomes independent of the average protein concentration and the quantified variability is then significantly larger than the one expected on the basis of the two-stage model. This effect does not seem specific to the type of protein, as all the highly expressed proteins are similarly impacted; therefore, a possible gene-specific phenomenon (such as the variability induced by the regulation of the gene) seems unlikely to explain the additional noise observed. After having ruled out several possibilities, the authors then proposed in their study cell-scale phenomena as possible explanations for this shift, in particular the fluctuations in the availability of RNA-polymerases and ribosomes in the production of different proteins. But other cell-scale mechanisms can also contribute significantly to protein variability, for example the partitioning that occurs at division, in which each compound (mRNA or protein) goes to either of the two daughter cells [10–12], or the gene replication event in which the transcription rate is doubled at some point in the cell cycle [12]. Finally, some assumptions considered in the two-stage model are questionable when applied in the context of Taniguchi et al., for example the fact that a death process acting on the proteins models the dilution effect due to cell volume growth (see [13]).

The study of Taniguchi et al. gives an extensive set of measurements for the majority of *E. coli* genes. This turns out to be very useful to link theoretical models with experimental data, not only to determine the parameters of the models, but also to compare the predictions of these models to experimental results. This was developed by Taniguchi et al. for the two-stage model, in which they concluded this classic model misses some cell-scale mechanisms to fully reflect experimental protein variability observed. Yet they did not try to develop models that include cell-scale mechanisms to definitely corroborate their hypothesis.

To tackle this limitation, we propose in this paper a model that integrates several cellular mechanisms which are not present in the two-stage model, and which are usually regarded as possible contributors to the variability of proteins. To set the model parameters, we used the measurements of 841 genes provided by Taniguchi et al.; those for which both the average mRNA and protein concentration have been measured. The predictions of analytical formulas or simulations using these parameters can then reveal and quantify the contribution of each of the cell mechanisms considered to the variability in gene expression.

In the next subsection, we present a review of the classic two-stage model as it is broadly used in the literature. We will explicitly present its limitations in regard to cell-scale mechanisms that are suspected to play a definitive role in protein variability. We then present aspects that need to be changed in order to represent these cell-scale mechanisms and that make it possible a quantitative comparison on their effect on protein expression. The Results section presents the predictions and results in three steps: we successively study 1) the effect on protein variance of random partition at division, 2) gene replication, and 3) fluctuations in the availability of RNA-polymerases and ribosomes. In the Discussion section, we discuss the comparison with the variability predicted with our models to those experimentally observed. We will show that even if some mechanisms significantly add variability to the system, it is not enough to explain the variance of highly expressed proteins observed experimentally; contrary to what has been suggested by Taniguchi et al. We will also discuss some phenomena not modeled that could impact the protein variability. The Materials and Methods section exhaustively describes our complete model, derived from the two-stage model, and the procedure used to analyze it.

### Limitations of the two-stage model

We based our approach on one of the simplest classic stochastic models of protein production, the two-stage model [7–9, 14]. This model describes the production and degradation of mRNAs and proteins of one particular type. In contrast to a three-stage model [9], it considers a constitutive gene, as it does not integrate a regulatory stage at the transcription initiation level, and thus represents the expression of a constitutive gene. The additional noise observed in Taniguchi et al. uniformly impacts highly expressed proteins, and therefore seems to be the result of cell-scale phenomena. Even if gene-specific mechanisms such as gene regulation can have a large impact on protein variance (see [15, 16] for instance), we rather are looking at mechanisms at the scale of the cell to explain the feature observed in Taniguchi et al.

**Fig 1:**
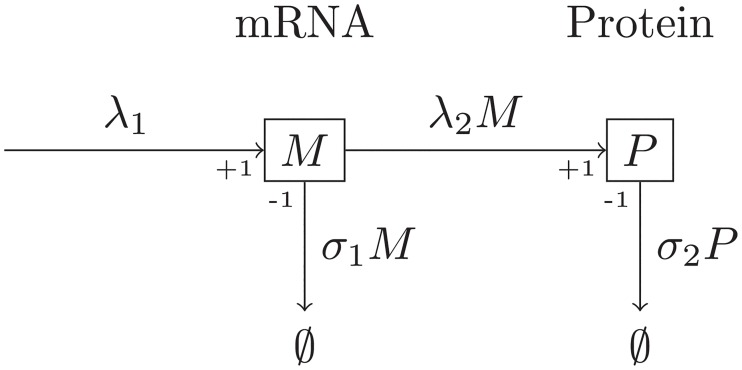
Classic two-stage model of one constitutive gene. *M* and *P* represent the respective numbers of mRNAs and proteins in a given cell. The parameter λ_1_ is the molecular-specific rate at which mRNAs are produced, λ_2_*M* is the rate at which proteins are created, *σ*_1_ indicates the molecular-specific mRNA degradation rate and *σ*_2_ is the rate at which protein leaves the cell (by the effect of volume growth and division).

The two-stage model represents the evolution of the random variables *M* and *P*, respectively representing the number of mRNAs and proteins associated with this gene inside a single cell (see Fig 1). It is important to note that the quantities considered in this model are integers. In Fig 1, *M* and *P* respectively denote the number of mRNAs and proteins inside the cell, but their concentrations are not explicit in the model, it does not incorporate the notion of cell volume.

Both the transcription and translation mechanisms are represented in the model, as well as mRNA degradation. There is also a protein decay mechanism that can either represent the degradation of the protein or the dilution effect induced by the growth of cell volume, since the proteolysis often occurs on a timescale much longer than the cell cycle (see [17]). All the events, like the production and degradation of mRNAs and proteins, are assumed to occur at exponentially distributed random times, the rates of which depend on the current state of the system.

The simplicity of the two-stage model is such that the identification of closed-form formulas for the mean and variance of mRNAs and proteins is possible (see [9]); moreover as it only represents mechanisms that are specific to protein production, the predicted variance can be naturally regarded as representative of the intrinsic noise, as has been done by Taniguchi et al. But the model lacks several features that are necessary for making quantitative comparisons with experimental results, and neglects several mechanisms that might have an impact on protein variance.

#### No representation of the volume

The fact that cell volume is not explicitly represented in the two-stage model (and those derived from it) does not seem to have been specifically highlighted in the literature. It appeared to us to be an important limitation of classic models (such as the two-stage model) for the three following intertwined reasons:

- Quantitative comparison with experimental concentrations. As previously stated, the two-stage model describes the evolution of the *number* of messengers and proteins in a single cell. It appears that the measurements of Taniguchi et al. represent concentrations, as this quantity is often more relevant than the numbers as it determines the speed of each reaction through the law of mass action. The direct quantitative comparison of means and variances obtained from the two-stage model to those obtained experimentally is not completely licit. In order to have a model for bacterial growth than can be compared with the experimental data, it is necessary to explicitly describe the volume, in order to represent concentrations in the cytosol, and not only numbers.
- Impact of volume evolution on concentration. In real cells, the simple growth of cell volume has a direct mechanical impact on compound concentrations through dilution. This effect of dilution of proteins is approximated in the two-stage model by a degradation process at exponentially distributed random times [9], whose rate *σ*_2_*P* depends on the protein number, the parameter *σ*_2_ being linked to the doubling time. But in reality, dilution is a different process and should be represented as an explicit volume increase rather than a protein disappearance rate. Furthermore, the effect of volume growth on messenger concentration is completely neglected in the two-stage model (due to their quick degradation).
- Impact of volume evolution on reaction rates. Volume growth also has another impact on protein production, as it tends to slow down the reaction rates by diminishing the reactant concentrations in the law of mass action. This will have important consequences, as we will explicitly represent RNA-polymerases and ribosomes (see below), and the volume growth will impact their respective concentrations and thus global protein production rate.

#### No partition at division

The two-stage model represents the expression of one gene in a single cell, so protein decay represents the tendency of proteins to disappear from the cell through the effect of division. In reality, division is a relatively sudden event that partitions both mRNAs and proteins in the two daughter cells.

#### No gene replication

The two-stage model does not consider genetic replication events, implicitly assuming that the gene promoter number is the same, thus keeping the rate of transcription λ_1_ constant. In reality, the activity of the gene is linked to its promoter concentration, which tends to decrease with the cell cycle as the volume increases, until the promoter is replicated. At this point, the rate of transcription is doubled, thus possibly inducing a transcription burst.

#### Constant availability of RNA-polymerases and ribosomes

RNA-polymerases and ribosomes are needed in protein production for transcription and translation respectively. As these resources are shared among all the productions of all the different proteins, their availability fluctuates over time. Since the two-stage model is a gene-centered model (it represents the expression of only one gene), it is not able to represent the competitive interactions between the different production processes for available RNA-polymerases and ribosomes. In particular, the mRNA production rate λ_1_ and protein production rate per mRNA λ_2_ are constant, as if the concentrations of available RNA-polymerases and ribosomes remain constant throughout the cell cycle.

Several of these external mechanisms have been experimentally and theoretically tackled in the literature: random partitioning at division in [11, 18, 19], gene replication and gene dosage in [18, 20–25] or fluctuations in the availability of ribosomes in [26]). Nonetheless, all these papers concentrate at one mechanisms at a time and there is no global perspective with quantitative comparison on the respective impact of these mechanisms. Questions like “What are the respective quantitative effects on protein fluctuations of random partitioning, gene replication or the sharing of ribosomes and RNA polymerases when comparing to the intrinsic noise due to the protein production mechanism itself?” still need to be handled. Theoretical models, associated with biologically relevant parameters will allow us to answer this kind of challenging questions.

During the writing of the current article, two studies [27, 28] were published: they both have a similar global approach that tend to represent whole cell cycle with DNA replication and division. Yet our study model differently several key aspects of the protein productions. In Lin and Amir [28], the sequestration of RNA polymerases and ribosome during the elongation are not explicitly modelled; like many of the previous studied cited above, the rate of each reaction only depends on the total number of compounds rather their concentration thus do not take into account the dilution effect (for instance, all things equal otherwise, the activity of a single promoter will tend to decrease as the cell volume grows, simply by dilution). In Thomas et al. [27], has a broader approach by gathering proteins into groups (Transporters, Enzymes, Housekeeping, Ribosomes, etc.) and the rate of productions are specific to these groups; if global cell mechanisms are more integrated in this modelling, our approach has a gene specific precision that can be used to see the effect of different cell mechanisms on the whole diversity of different proteins. In both case, neither of these study has a direct quantitative comparison with experimental measures; as our study fit its parameters and compare directly its predictions to the experimental measures of Taniguchi et al. [6].

## Results

In the Materials and Methods section, we describe in detail our approach that considers models that integrates the features that are missing in the two-stage model previously described. In order to determine the relative impact of each of these aspects, we have proceeded in successive steps of increasing complexity. Below is presented the results concerning three intermediate models that successively incorporate a specific feature; the impact of each of these features on protein variance will be studied one at a time. The three intermediate models are the following:

- The first intermediate model only considers the growth of the cell and study the impact of the partitioning at division. In particular, each gene concentration, as well as the free RNA-polymerase and ribosome concentrations, are considered constant during the whole cell cycle. It focuses on the effect of the partition at division on protein variance.
- The second intermediate model then study the effect of gene replication, while still considering cell volume growth and partitions at division but keeping free RNA-polymerase and ribosome concentrations as constant.
- Then is considered the complete model, with fluctuating free RNA-polymerases and ribosomes, replication, volume growth and partitions at division.

The next three subsections will present each of these models one at a time. For each of the models, the parameters are fitted in order to correspond to each of the genes of Taniguchi et al. and the protein concentration variance using the procedure described in Materials and Methods.

### Impact of random partitioning

The first intermediate model focuses on the effect of random partitioning. Fluctuations in the availability of RNA-polymerases and ribosomes, as well as the gene replication is therefore not considered. As the global sharing of ribosomes and RNA-polymerases are not represented, it results in a model where there are no interactions between the productions of different proteins; each production can be considered separately. It is a gene-centered model and one can focus on the production of one particular type of protein; *M* and *P* being respectively the number of mRNAs and proteins of this type.

**Fig 2:**
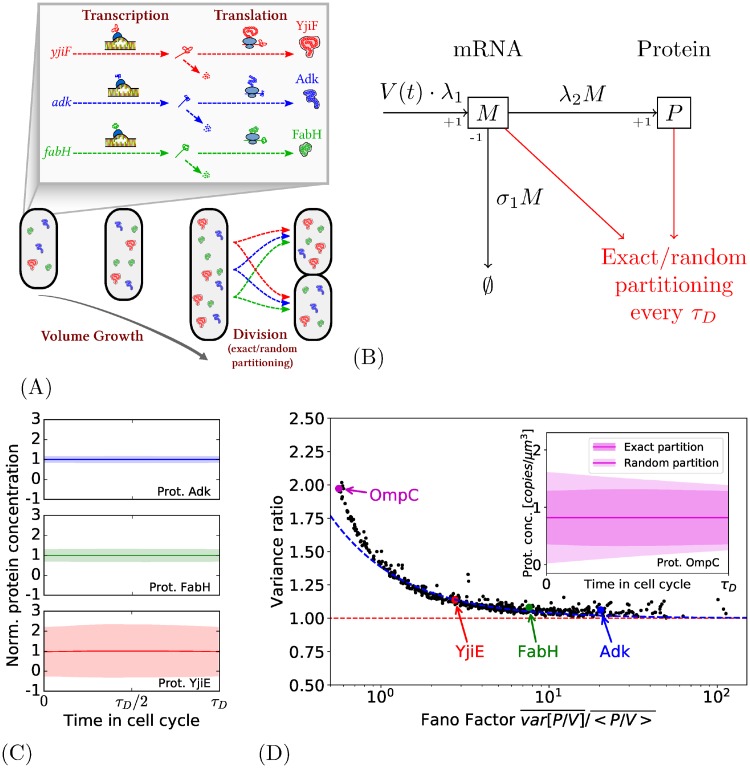
Intermediate model with volume growth and partition at division. (A): The model considers transcription, translation, volume growth, and partitioning at division. (B): Model of production for one type of protein. The parameters λ_1_, *σ*_1_ and λ_2_ are specific to the type of protein. The gene concentration is considered as constant, giving a rate of transcription proportional to cell volume. (C): The evolution of the normalized protein concentration during the cell cycle for three proteins. The central thick line represents the average production during the cell cycle and the colored area the standard deviation. (D) Main: For each type of protein, the variance in the case of random partition divided by the variance in the case of exact partition as a function of the Fano Factor (see main text and Section 1.5 of S1 Appendix). The proportion of variance attributed to the effect of the random partitioning globally follows the prediction of the simplified model in blue dashed line (see Section 1.5 of S1 Appendix). Section Inset: Comparison between the two partitions for the protein OmpC. The thick line is the average protein concentration during the cell cycle and the colored areas correspond to the standard deviation in the two cases.

Fig 2 depicts this intermediate model. The parameter λ_1_ of transcription (resp. λ_2_ for the translation) implicitly includes some aspects specific to the gene (the promoter–polymerase affinity, for instance) and the effective constant concentration of free RNA-polymerases (resp. free ribosomes). As the gene is in constant concentration, the rate of mRNA creation increases alongside the volume; it is therefore equal to λ_1_*V*(*t*), with *V*(*t*) the volume of the cell at time *t*. Hence, the rate of mRNA production per volume unit remains constant.

For this intermediate model, a deterministic volume growth is considered. Based on experimental results (see [29]), we represent the growth of the bacteria volume as exponential. For this model, if *t* is the time spent since the last division, the volume is given by

$$\begin{array}{c} V\left(t\right)=V_{0}2^{t/\tau_{D}}, \end{array}$$

with *V*_0_ being the typical size of a cell at birth and *τ*_*D*_ the duration of the cell cycle. The explicit description of the volume leads to consider the concentration of mRNA *M*(*t*)/*V*(*t*) and proteins *P*(*t*)/*V*(*t*) at any time *t* of the cell cycle.

Note that in this model and the subsequent ones, we interpret the division mechanism as a “sizer” model: the division occurs when the cell reaches the volume 2*V*_0_ [29–32]. Even if in the present model, the exact dependence of the volume to the cell cycle time can bring us to either interpret it as a “timer” (occurring at time *τ*_*D*_) or a “sizer” (occurring when reaching volume 2*V*_0_); the “sizer” interpretation will be preferred as it is the one that ensures cell size stability and has been shown to be a good first approximation to explain experimental cell size distributions [32, 33]. The last intermediate model will later explicitly use a “sizer” mechanism.

In this first model, we study the effect of partitioning on protein variance. Two mechanisms of segregating compounds at division are compared, either an exact or a random partitioning.

- for the exact partitioning, the number of proteins and mRNAs at division are equally allocated between the two daughter cells. Clearly, this mechanism does not have an impact on the variance of the mRNA and protein concentrations;
- for the random partitioning at division, each mRNA and protein has an equal chance to be in either one of the two daughter cells (so that with probability 1/2, they are in the next cell of interest). An additional variability, due to this random allocation, should be therefore added in this case.

As depicted in Materials and Methods, we first perform a theoretical analysis of this model in order to predict its mean concentrations of mRNA and protein averaged over the cell cycle: respectively $\bar{\langle M/V\rangle }$ and $\bar{\langle P/V\rangle }$ as they are defined in Eq (6) of the Materials and Methods section. In this model, the mean concentration 〈*M*(*t*)/*V*(*t*)〉 and 〈*P*(*t*)/*V*(*t*)〉 *during* the cell cycle remain constant. We then have that for any time *t* of the cell cycle:

$$\begin{array}{c} \langle M(t)/V(t)\rangle =\bar{\left\langle M/V\right\rangle }=\frac{\lambda_{1}\tau_{D}}{\sigma_{1}\tau_{D}+\text{log}\;2}\\ \;\;\;\;\;\text{and}\;\langle P(t)/V(t)\rangle =\bar{\left\langle P/V\right\rangle }=\frac{\lambda_{2}\tau_{D}}{\text{log}\;2}\cdot \frac{\lambda_{1}\tau_{D}}{\sigma_{1}\tau_{D}+\text{log}\;2}. \end{array}$$ (1)

Proofs of these formulas can be found in Sections 1.1.1 and 1.1.2 of S1 Appendix. The mRNA degradation times and the time of cell cycles are directly measured in [6], thus setting the parameters *σ*_1_ and *τ*_*D*_. With that, these previous formulas make it possible, for every gene considered in [6], to set the parameters λ_1_ and λ_2_ in order to have an average production that corresponds to those experimentally measured. This gives a series of parameters corresponding to a representative sample of real bacterial genes (more details in Section 1.2 of S1 Appendix). As described in Section 1.3 of S1 Appendix, simulations are performed using an algorithm derived from Gillespie method in order to determine the evolution of protein concentration across the cell cycle and determine its variance averaged over the cell cycle $\bar{\text{Var}}[P/V]$—as it is defined in Eq (7) of Materials and Methods section. We then check that the average protein and mRNA concentrations correspond to those experimentally measured (see S1 Fig).

Fig 2C shows results of exact simulations (using Gillepsie-related algorithm): the figures show the profile of protein concentration during the cell cycle for three representative genes (*adk*, *fabH* and *yjiE*) which are respectively highly, moderately and lowly expressed. As predicted theoretically, the mean concentration 〈*P*(*t*)/*V*(*t*)〉 does not change across the cell cycle (it is due to the fact that the gene concentration remains constant).

Fig 2D shows the proportion of variance that is added with the introduction of random partitioning. It appears that for all genes, their protein variance indeed increases with random partition, up to be doubled for some genes (like for the protein OmpC whose profile is shown in the inset, where the average production remains constant in both cases, but the random partition increases protein variance at the beginning of the cell cycle.).

The *x*-axis of Fig 2D is somewhat unusual as it is the protein Fano Factor, defined as $\bar{\text{Var}}[P/V]/\bar{\langle P/V\rangle }$. It is used because the proportion of variance added by the random partition shows a remarkable clear dependence to the Fano Factor: proteins with a low Fano Factor are particularly more impacted. Note that rare proteins also tend to have low Fano factor (see S1(B) Fig), the global tendency remains the same with having the average production as an x-axis, even if this dependence is less strong (see S1(C) Fig).

In order to theoretically explain this clear dependence on the Fano factor, another simplified model, that focuses only on the partition effect without considering volume growth, has been analyzed (details in Section 1.5 of S1 Appendix). Its predictions are shown in blue dotted line of the main Fig 2D. It globally predicts the proportion of noise that can be attributed to the random partitioning. It confirms that this effect is only significant for proteins with very low Fano factor.

We also want to decompose the protein concentration variance in the same way as it is done experimentally using the dual reporter technique [1] where are observed correlated and non-correlated variances between the expression of two proteins with identical promoters: the part of the variance that is not correlated between the two protein expressions is interpreted as solely due to the variability of the production mechanism itself; while the correlated variance is due to the common environment in the cell of the two genes (such as the cell volume, the number of available RNA polymerases, etc.) that influences equally the two productions. Hilfinger and Paulson [34] showed that the theoretical counterpart of such decomposition is the environmental state decomposition of the variance (see details in Materials and methods). With the environmental state decomposition, the protein variance $\bar{\text{Var}}[P/V]$ can be decomposed into the two terms $\bar{{\text{Var}}_{int}}[P/V]$ and $\bar{{\text{Var}}_{ext}}[P/V]$ that would respectively represent the intrinsic (uncorrelated variance in the dual reporter technique) and extrinsic contribution (correlated variance in the dual reporter technique) to protein variance. A general formula for this decomposition can be found in the Materials and Methods, but in the current model, as the only external environment considered is the cell cycle (represented by the cell volume *V*), the docomposition of the variance of each protein $\bar{\text{Var}}[P/V]$ is the sum of

$$\begin{array}{c} \bar{{\text{Var}}_{int}}[P/V]=\frac{1}{\tau_{D}}\int_{0}^{\tau_{D}}\text{Var}[P(t)/V(t)]dt, \end{array}$$ (2)

$$\begin{array}{c} \bar{{\text{Var}}_{ext}}[P/V]=\frac{1}{\tau_{D}}\int_{0}^{\tau_{D}}{\left\langle P(t)/V(t)\right\rangle }^{2}dt-{\bar{\left\langle P/V\right\rangle }}^{2} \end{array}$$ (3)

(see Section 1.4 of S1 Appendix for more details).

In this intermediate model, even with random partitioning, such a decomposition shows surprisingly no external contribution $\bar{{\text{Var}}_{ext}}[P/V]$ for every protein (since the protein concentration *P*(*t*)/*V*(*t*) remains constant across the cell cycle). It is therefore remarkable that this decomposition only captures a part of what is generally accepted as the extrinsic noise. We verified this decomposition by a simulation that reproduces the dual reporter technique: we considered the expression of two identical promoters, with the same parameters, in the same cell. The covariance of the two protein concentrations measured in the simulation is much smaller than the variance of each protein concentration (see Section 1.4 in S1 Appendix and S1(D) Fig).

### Impact of gene replication

**Fig 3:**
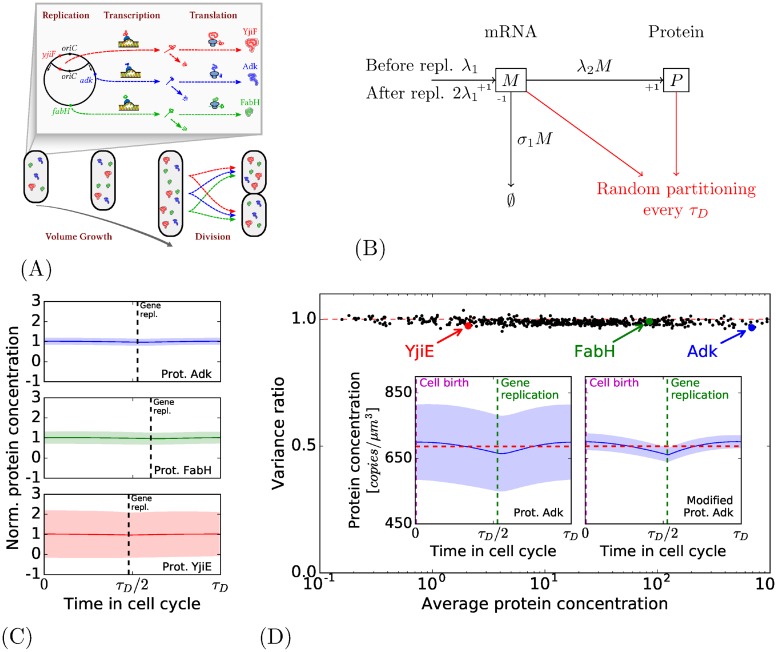
Intermediate model with cell cycle and gene replication. (A): Features of the intermediate model. The model now takes into account replication. (B): The production of one particular type of protein. The number of mRNAs and proteins are respectively *M* and *P*; the difference with the previous model is the introduction of replication at a time *τ*_*R*_ after the cell birth where the transcription rate is doubled. (C): The evolution of the normalized protein concentration during the cell cycle for three proteins. The central thick line and the colored areas represent the same quantities as in Fig 2C. (D) Main: for each type of protein, protein variance of the previous model (gene in constant concentration and random partitioning) divided by the one in this model. The replication paradoxically tends to slightly diminish the fluctuations of the concentration. Insight: on the left, the concentration through the cell cycle for the protein Adk (a close-up of the one presented in Fig 3C); on the right, profile of a modified version of Adk with parameters chosen in order to minimize the variance (see main text).

Taking back the model previously described with volume growth and random partitioning at division, we introduce the notion of gene replication. As in the case of the slowing growing bacteria of [6], we consider only one DNA replication per cell cycle. The gene is represented for now on as an entity that is replicated at some instant *τ*_*R*_ of the cell cycle, hence doubling the transcription rate (*τ*_*R*_ is determined depending on the position of the gene on the chromosome, see Materials and methods): before a time *τ*_*R*_ after the cell birth, the rate of transcription will be λ_1_, after the time *τ*_*R*_, the rate of transcription is doubled (see Fig 3B).

The mean of mRNAs concentration can be determined at any moment of the cell cycle. At any time *t* in the cell cycle (with 0 ≤ *t* < *τ*_*D*_), it is given by,

$$\begin{array}{c} \langle M(t)/V(t)\rangle =\frac{\lambda_{1}}{\sigma_{1}V\left(t\right)}[1-\frac{e^{-\left(t+\tau_{D}-\tau_{R}\right)\sigma_{1}}}{2-e^{-\tau_{D}\sigma_{1}}}+1_{\left\{t\geq \tau_{R}\right\}}(1-e^{-\left(t-\tau_{R}\right)\sigma_{1}})], \end{array}$$ (4)

with **1**_{*x*≥*y*}_ being the Heaviside function (**1**_{*x*≥*y*}_ = 1 if *x* ≥ *y* and 0 otherwise). One can refer to Section 2.1.1 of S1 Appendix for the proof.

Similarly, the mean of protein concentration can also be explicitly determined. Before the replication, if *t* indicates the time after the birth of the cell (i.e. 0 ≤ *t* < *τ*_*R*_), we can determine the mean of *P*(*t*)/*V*(*t*) as a function of the mean of *M*(0) and *P*(0). Similarly, after the replication, the mean of *P*(*t* + *τ*_*R*_)/*V*(*t* + *τ*_*R*_) (with *t* such as 0 ≤ *t* < *τ*_*D*_ − *τ*_*R*_) is known as a function of the mean of *M*(*τ*_*R*_) and *P*(*τ*_*R*_). They are given by the formula,

$$\begin{array}{c} \langle P(t+\tau )/V(t+\tau )\rangle =\frac{1}{V(t+\tau )}[\langle P(\tau )\rangle +\frac{\eta \lambda_{1}}{\sigma_{1}}\lambda_{2}t+(\langle M(\tau )\rangle -\frac{\eta \lambda_{1}}{\sigma_{1}})\frac{1-e^{-\sigma_{1}t}}{\sigma_{1}}], \end{array}$$ (5)

with *τ* = 0 and *η* = 1 for the case before the replication and *τ* = *τ*_*R*_ and *η* = 2 for the case after the replication. At steady state we are able to determine explicit values for 〈*P*(0)〉 and 〈*P*(*τ*_*R*_〉 as a function of the parameters (see Section 2.1.2 of S1 Appendix).

As for the previous model, Eqs (4) and (5) make it possible, for every gene experimentally measured in [6], to give a set of parameters λ_1_, *σ*_1_ and λ_2_ that corresponds to it (Section 2.2 of S1 Appendix and S2(A) Fig). To determine the variance of protein concentration, simulations can be performed, but it is noticeable that we also managed to have formulas for mRNA and protein variances. These formulas greatly simplify the analysis of this intermediate model (see Sections 2.1.1 and 2.1.2 of S1 Appendix).

Fig 3C presents the normalized profiles for three different proteins with different replication times in the cell cycle: their normalized average concentration (thick line) and their normalized standard deviation (colored area) during the cell cycle are shown. Globally, these profiles present little changes compared to the ones of the previous model (Fig 2C). In the left figure of the inset of Fig 3D is shown the profile of the protein Adk (a closeup of the first profile of Fig 3C). It appears that the mean concentration at any given time *t* of the cell cycle 〈*P*(*t*)/*V*(*t*)〉 (the thick line of the profile) is not constant during the cell cycle, as it was the case in the model of Fig 2. The curve of 〈*P*(*t*)/*V*(*t*)〉 fluctuates around 2% of the global average protein concentration $\bar{\langle P/V\rangle }$.

The main Fig 3D shows the effect of replication on the variance: it represents the ratio of protein variance between the previous model (with genes in constant concentration and random partitioning) and the current model with gene replication. For all the genes, the variances predicted show little difference from the previous intermediate model. The ratio is even surprisingly slightly above one for many genes, indicating that for these genes the replication tends to reduce the variance.

As for the previous intermediate model, we can use the environmental state decomposition to separate the part of variance $\bar{{\text{Var}}_{int}}[P/V]$ (defined in Eq (2)) specific to the gene expression and $\bar{{\text{Var}}_{ext}}[P/V]$ (defined in Eq (3)) attributed to cell cycle fluctuations. As the mean concentration, 〈*P*(*t*)/*V*(*t*)〉 is no longer constant during the cell cycle $\bar{{\text{Var}}_{ext}}[P/V]$ is no longer null. Yet it appears that $\bar{{\text{Var}}_{ext}}[P/V]$ only represents a very small part of the global variance $\bar{\text{Var}}[P/V]$ (for 99% of the genes, it represents less than 2%). For this intermediate model, the extrinsic contribution of DNA replication computed with this decomposition is small.

Using the analytical formula of protein variance (Section 2.1.2 of S1 Appendix) and by performing variations on certain parameters, we can analyze the effect of several aspects on protein variance in this model. By considering a given protein (protein Adk), we subsequently modified different parameters while making sure that the average protein concentration remain unchanged, to see how each of these changes impact the protein variance:

- Changing the position of the gene on the chromosome (specifically changing *τ*_*R*_ and slightly adapting the gene activation rate λ_1_ to keep the same average mRNA concentration)
- Changing the mRNA lifetime (by increasing the gene activity λ_1_ and increasing the mRNA degradation rate *σ*_1_ so that it keeps the same average mRNA concentration)
- Changing the mRNA number (by increasing the gene activity λ_1_ while decreasing the mRNA activity λ_2_ so that it keeps the same average protein concentration)

Results are shown in S2 Fig(C) of S1 Appendix. Changing the gene position have almost no impact on global protein variance $\bar{\text{Var}}[P/V]$. The effect of mRNA lifetime is more noticeable as a shorter mRNA lifetime can diminish protein variance at most about 40%. The mRNA number seems to have the most important effect on protein production: for the same average protein concentration, having more mRNAs greatly diminish protein variance; such effect has been experimentally observed [2, 35]. This can be interpreted as lower bursting effect in protein production: as it is known that mRNAs in few copies with large activity display a protein production with large bursts, conversely a large number of mRNAs less active leads to a more stable protein production.

The right insight of S2 Fig(C) of S1 Appendix shows an example of such a protein with reduced variance: this protein is based on Adk, the protein average production is the same but there are ten times more mRNAs, with a ten times shorter lifetime. The variance is indeed reduced but with the cost of the production of additional mRNAs. Yet, even in this case, the protein expression is not strongly cycle-dependent (see inset of S2 Fig(D) of S1 Appendix); in particular, this profile is not precise enough to be used as a “trigger” for periodic cell events (such as DNA replication initiation, or partition at division): the evolution of the protein concentration across the cell cycle is not precise enough to robustly distinguish different phases of the cell cycle.

### Impact of the sharing of RNA-polymerases and ribosomes

We now consider the complete model, which includes a limited amount of RNA-polymerases and ribosomes: the model is explained in detail in the Materials and Methods. Now, RNA-polymerases and ribosomes are explicitly represented in the model and each of these macromolecules is considered either allocated (i.e. sequestered on a gene if it is an RNA-polymerase, or on an mRNA if it is a ribosome), or free (i.e. either moving freely in the cytoplasm or, in the case of RNA-polymerases, potentially non-specifically sliding on the DNA).

The previous intermediate models were “gene-centered”: each class of proteins was considered independently from each other. The common sharing of RNA-polymerases and ribosomes is an additional key feature that leads us to investigate a multi-protein model where all the genes of the bacteria are considered altogether. For each type of protein *i*, we denote by *G*_*i*_(*t*), *M*_*i*_(*t*) and *P*_*i*_(*t*) respectively the number of gene copies, of messengers and of proteins at time *t* in the cell cycle. For each gene *i*, *E*_*Y*,*i*_(*t*) is the number of RNA-polymerases sequestered on the *i*-th gene for transcription and *E*_*R*,*i*_(*t*) is the number of ribosomes sequestered on an mRNA of type *i* for translation. The non-allocated RNA-polymerases and ribosomes are respectively denoted by *F*_*Y*_(*t*) and *F*_*R*_(*t*). In a first step, we have considered that the gene pool of [6] (841 genes with their mRNA and protein expression measured) would represent the whole genome. We will later see that the addition of new genes does not change significantly our results.

New ribosomes and RNA-polymerases are added to the system as cell volume increases: in a first step, these macromolecules are regularly added such as their total concentration in the cell remains constant during the cell cycle; we will later consider a more realistic way to represent RNA-polymerase and ribosome production.

The previous intermediate models represented the production of a specific type of protein immersed into a “background environment” where the cell grows and divides, the current model includes simultaneously all the genes altogether. In this model, as we are on the scale of the whole cell, we would like to model the impact of global protein production on the cell growth. We therefore can no longer consider that the production of each type of protein has no effect on the global performance of the cell. The volume *V*(*t*) depends now on the global production of proteins, and it is not an independent and deterministic feature anymore. As the density of cell components tends to be constant in real-life experiment [36] and proteins represent more than half of the dry mass of the cell [37], the model considers the volume as proportional to the current total mass of proteins in the cell. The mass of each protein is given by the length of its peptide chain (see Section 3.2.1 of S1 Appendix for an exhaustive description of the model). Like in the previous model, division occurs when the cell reaches the volume 2*V*_0_ (making it a “sizer” model).

The processes of mRNA and protein productions are both separated in two parts: the binding and initiation on one side, and the elongation and termination on the other side. The rate at which an RNA-polymerase is sequestered on a gene of type *i* at time *t* depends on the copy number of the *i*-th gene *G*_*i*_(*t*), the free RNA-polymerase concentration *F*_*Y*_(*t*)/*V*(*t*) and a parameter λ_1,*i*_ specific to the gene that takes into account the RNA-polymerase–promoter affinity. The elongation rate of each mRNA only depends on the average transcription speed and the length of the gene. The mechanism for translation is similar.

As this model is more complex than the previous ones, the complete analytical description of mRNA and protein dynamics seems to be out of reach. To address this problem, we try to predict the average behavior of this model using a system of ordinary differential equations (ODEs). We used the predictions of these equations to fix the parameters. An *a posteriori* validation has been made to check that this system of ODEs well predicts the average behavior of the stochastic model (see Section 3.2.1 of S1 Appendix for more details on the procedure).

**Fig 4:**
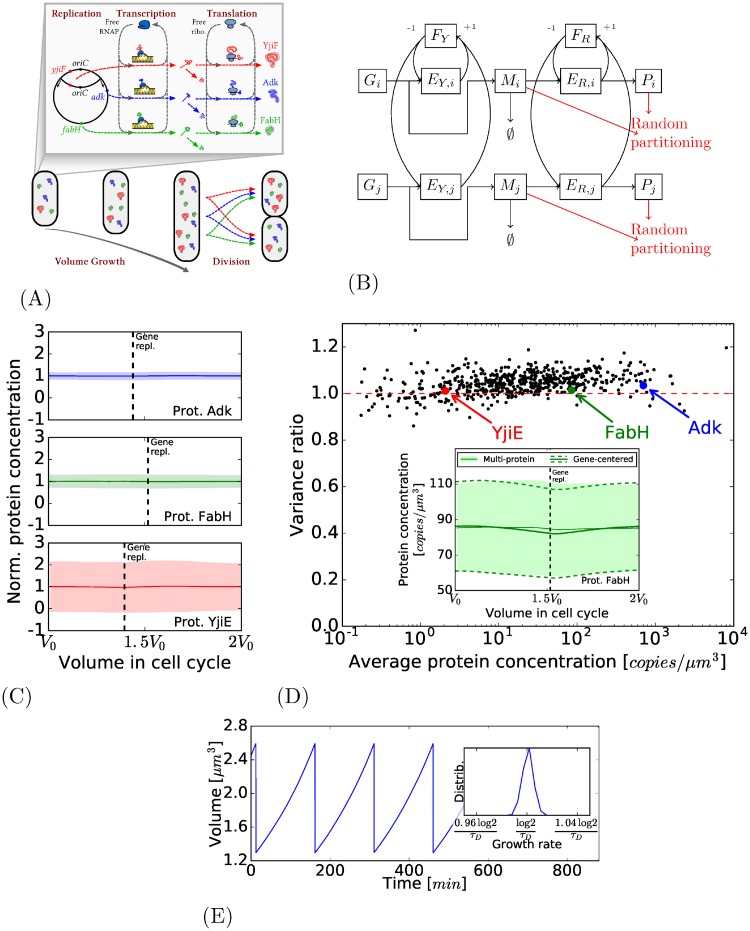
Complete model. (A): The model now considers the sharing of RNA-polymerases and ribosomes between the different productions. (B): The model of production of all proteins. The *i*-th gene is associated with one particular type of mRNA (whose number is *M*_*i*_) and proteins (whose number is *P*_*i*_). The number of free RNA-polymerases (resp. ribosomes) is *F*_*Y*_ (resp. *F*_*R*_), the number of those sequestered on the *i*-th gene is *E*_*Y*,*i*_ (resp. *E*_*R*,*i*_). (C): The evolution of the normalized protein concentration during the cell cycle for three proteins. The central thick line and the colored areas represent the same quantities as in Fig 2C. (D) Main: variance in the previous model with replication divided by the variance in this model with sharing of RNA-polymerases and ribosomes. Insight: the concentration through the cell cycle for the protein FabH (a close-up of the one presented in Fig 4C). (E) Main: A simulation sample that shows that cell volume grows exponentially from around *V*_0_ up to around 2*V*_0_. Insight: The growth rates of the simulation are centered around the expected growth rate log 2/*τ*_*D*_.

In Fig 4E is shown an example of the cell volume evolution in a simulation. It appears that the volume seems to grow exponentially during the cell cycle. The number of available ribosomes and RNA-polymerases also changes rapidly, of the order of the second for the RNA-polymerases, and of the order of one tenth of a second for the ribosomes (see S3 Fig(C) and S3 Fig(D) of S1 Appendix).

Fig 4D compares the results of the simulations with the previous intermediate model with gene replication and random partitioning. It shows that, for 90% of the genes, the interactions between protein productions only represent at most 10% of variability. In inset is shown the example of the protein FabH profile during the cell cycle, showing that sharing of RNA-polymerases and ribosomes introduces little change.

We also analyze the model using the environmental state decomposition. Two genes of the dual reporter technique would undergo the same volume growth with the same evolution of free RNA-polymerases and ribosomes; as a consequence, the common cellular environment on which the decomposition is operated now includes the concentration of free RNA-polymerases and ribosomes (in addition to the cell cycle). Details can be found in Section 3.7 of S1 Appendix. It appears that for all the genes, the extrinsic contribution of the variance $\bar{{\text{Var}}_{ext}}[P_{i}/V]$ represents only a very small portion of $\bar{\text{Var}}[P_{i}/V]$ (for 99% of the cells, the ratio $\bar{{\text{Var}}_{ext}}[P_{i}/V]/\bar{\text{Var}}[P_{i}/V]$ represents less than 1%).

We compared these simulation results with a simplified theoretical model: Section 3.6 of S1 Appendix presents a multi-protein model, that is inspired by the one described in [26]. Even if it is a multi-protein model as it represents the expression of a large number of genes altogether, it is a simpler model than the one presented here as it considers separately transcription and translation, and it does not consider neither volume growth, partitioning at division, nor DNA replication. We show that the predicted distributions of free RNA-polymerases (and ribosomes in the adapted model) fits well the one observed in our simulations (see S4 Fig). As we will see in the discussion, this good correspondence between the models would suggest that the mean-field mathematical properties proven for the simplified model could be applied to our complete model.

### Model and parameter sensitivity

The complete model supposes a series of modeling and parameter choices that might legitimately influence protein production. We have analyzed several of these aspects and have shown that they do not appear to significantly change the results previously presented.

#### Quantity of free RNA-polymerases and ribosomes

The average concentration of free RNA-polymerases and ribosomes in a cell cannot be deduced from Taniguchi et al. They are nevertheless needed to estimate the parameters of our model, see Section 3.3 of S1 Appendix. Globally, one can expect to have a low concentration of free ribosomes and a higher concentration of free RNA-polymerases, see Section 3.5.1 of S1 Appendix. But precise numbers seem to be difficult to obtain. We therefore perform several simulations with different values for these concentrations (for each macromolecule, a concentration taking successively 1, 10, 100 and 1000 copies/μm^3^), without significant changes. See S4 Fig and Section 3.5 of S1 Appendix for details.

#### Additional genes

**Fig 5:**
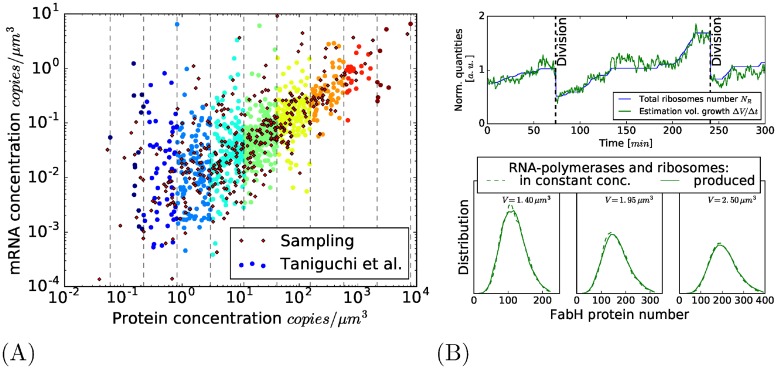
Model and parameter sensitivity. (A): Creation of additional genes by sampling of mRNA and protein concentrations in accordance to their correlation (see Section 3.6.1 of S1 Appendix for details on the procedure). (B): A variation on the complete model where the RNA-polymerases and ribosomes are produced by the cell. Above: clear correlation between the total number of ribosomes and the estimation of volume growth. Below: comparison between the two versions of the model (with or without production of RNA-polymerases and ribosomes); the distribution of protein number distribution of FabH protein for cells of different volumes in both cases.

As previously said, to perform our first simulations, only 841 genes from which the average mRNA and protein concentration have been measured in Tanuguchi et al. are considered. To have a pool of proteins that might represent a global gene expression in *E. coli*, we studied the case of a simulation with a set of genes that represent about 2000 genes, more in accordance to the expected number of genes expressed in a growing condition. To propose realistic parameters for these fictional genes, we sample them according to different empirical distributions observed in the empirical data, and also by taking into account the possible correlations observed (the correlation that exists between the average mRNA and protein concentration for instance). See Fig 5A and Section 3.6.1 of S1 Appendix. No changes in protein concentration variance is observed.

#### Non-specific binding of RNA-polymerases

It has been proposed that many of the RNA-polymerases are non-specifically bound on the DNA (see [38] for instance). We have done a simulation where RNA-polymerase can bind non-specifically on the DNA. When in this state, they are not available for the transcription. As previously it does not appear to change the protein expression behavior. See Section 3.6.3 of S1 Appendix.

#### Production of RNA-polymerases and ribosomes

The total amount of RNA-polymerases and ribosomes (whether free or not) were at first considered in constant concentration: the RNA-polymerases and ribosomes were added as cell volume increases. We have done a simulation that considers a way to represent their production to have a more realistic representation: both RNA-polymerases and ribosomes are produced as if they were one of the proteins of the system (this goal of the simulation is just to have an insight of the effect of RNA-polymerase and ribosome production, not to represent precisely their production mechanisms). The introduction of such mechanism indeed changes some aspects of the simulation: in particular, the growth of the cell is more erratic as it then directly correlated with the total number of ribosomes (see Fig 5B, above). But as the production of proteins increases with a higher number of ribosomes, so does the volume of the cell. In terms, of concentration, the induced fluctuations in the number of ribosomes have little impact in terms of protein concentration variance (see Fig 5B, below). See Section 3.6.2 of S1 Appendix for more details.

#### Precision in the division and DNA replication initiation timing

The initial simulation triggers DNA replication initiation and division when the cell reaches some volume. Yet fluctuations in the timing of division has been proposed to have an impact on the protein variance [25]. We investigate approximate models of division and of DNA replication initiation by introducing a volume-dependent rate of division as it is commonly used in the literature, see [29–32] (see Section 3.6.4 of S1 Appendix). As previously (when we investigated the effect of the production of RNA-polymerases and ribosomes), if the protein *number* is impacted, the protein *concentration* remain relatively unchanged due to the fact that fluctuations induced by uncertainty in the timing of division affect correlatively the protein number and the cell volume.

## Discussion

### Interpretation of the model predictions

The experimental data of Taniguchi et al. gave us the opportunity to systematically and quantitatively inspect the impact on protein variance of many cell mechanisms that are not often considered in stochastic models of protein production. The broad variety of genes experimentally measured in Taniguchi et al. has been a good opportunity for us to realistically test our models for a wide number of different genes, with different mRNA and protein concentrations, different mRNA lifetimes or gene position on the chromosome or gene length.

From this analysis, it appears that among the different features included in the model, the random partitioning has the most significant effect on the variance of protein concentration, especially for the less expressed proteins. We recover here one of the conclusions made in [27]. The gene replication induces little difference (it even tends, to a small extent, to reduce the variance in some cases); the important fluctuations of free RNA-polymerases and ribosomes have little impact on protein production, which does not fit the hypothesis made in [6]. It is confirmed by the environmental state decomposition, which separates the intrinsic and extrinsic contribution to protein variance (in an analog way as it is done with the dual reporter technique): the extrinsic contributions represent at most a few percents of the total variance.

We interpret the surprising little impact of sharing of RNA-polymerases and ribosomes on the proteins variance by noticing the similarities of our model with the one described in [26]. Indeed, as previously explained, the global behavior of free RNA-polymerases and ribosomes can be predicted by a simplified model derive from [26], where the RNA-polymerases and ribosomes are shared among the different productions. The main result of [26] is a mean-field theorem: as the number of genes increases, the production process of different types of proteins can be seen as independent production processes. The reason is that the dynamic of free RNA-polymerases and ribosomes is much faster than the production of mRNAs and proteins of one particular type. The rate at which an mRNA or protein is elongated only depends on the “local steady state” concentrations of free RNA-polymerases and ribosomes (a similar phenomenon can be found in [39]). Our model seems to display such mechanism: with a global sharing of RNA-polymerases and ribosomes by a large amount of protein productions, the dynamic of free RNA-polymerases and ribosomes is faster than the production of each mRNA and protein of each type. As a consequence, it is not surprising to see that this multi-protein model, which takes into account the production of all proteins displays little difference with the intermediate “gene-centered” model due to a mean-field effect.

### Comparison with experimental measures

In the end, we can compare the results of our models with the experiments. Firstly, one can remark that the profile of the mean production 〈*P*(*t*)/*V*(*t*)〉 (the plain line in Fig 3D is representative of all cells) during the cell cycle corresponds to the one observed experimentally in [22]. Furthermore, the maximum deviation of the average 〈*P*(*t*)/*V*(*t*)〉 around the global average protein $\bar{\langle P/V\rangle }$ (red dashed line in Fig 3D) is between 2% and 4% for all the proteins of our models, and Walker et al. measure such fluctuations also around 2% of the global average for genes at different positions on the chromosome (see Figure 1.d and Figure S6.b of [22]).

**Fig 6:**
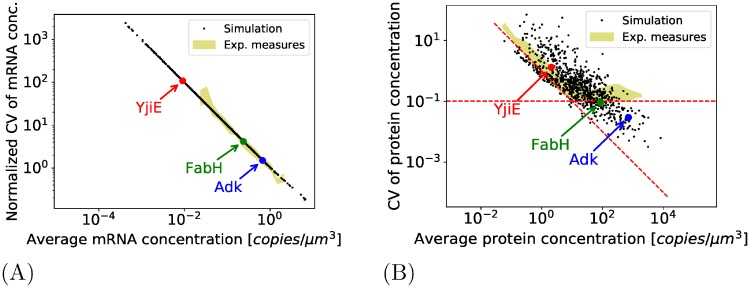
Comparison with experimental dataset of Taniguchi et al. (A): The normalized CV of mRNA concentration (defined as $\bar{\text{Var}}[M/V]/{\bar{\langle M/V\rangle }}^{2}$) for each gene predicted by the complete model (in yellow, the corresponding experimental measurements). (B): The CV of protein concentration for each gene predicted by the complete model (in yellow, the corresponding experimental measurements).

Secondly, we compared the global mRNA and protein fluctuations predicted in our models with those measured in Taniguchi et al. Fig 6A and 6B shows respectively, for every gene, the protein CV of mRNAs and proteins (resp. defined as $\bar{\text{Var}}[M/V]/{\bar{\langle M/V\rangle }}^{2}$ and $\bar{\text{Var}}[P/V]/{\bar{\langle P/V\rangle }}^{2}$) against their respective average concentration (resp. $\bar{\langle M/V\rangle }$ and $\bar{\langle P/V\rangle }$); it is compared in both cases with the same results obtained experimentally (respectively corresponding to measurements shown in Figures 2.D and 2.B of [6]). For the mRNAs (Fig 6A), the noise globally scales inversely the average mRNA concentration. Experimental measurements in [6]—made using the FISH technique for 137 of the highest expressed mRNAs—show a similar tendency (the CVs are normalized on the figure because the uncertainty in the cell volume at birth can introduce a shift, but the shape of the normalized CV of both experimental and simulated CV remains exactly the same regardless of this effect). For the protein CV (Fig 6B), it appears that the noise approximately scales inversely the average protein concentration like in the first “intrinsic noise” regime of [6]. But unlike in the [6] experiment, there is no lower plateau for highly expressed proteins: for the highest produced proteins, the CV should be in the order of 10^3^ fold higher than the one predicted. It confirms that the features considered here cannot correctly explain the noise observed experimentally.

We can propose different interpretations to explain the discrepancy between the predictions of the models and the experimental measures. For the biological processes not included in our models and that can have an impact on the variability, one can first mention the gene regulation as in our models, all the genes are considered as constitutive. The introduction of a gene regulation can indeed induce a large variability in protein concentration [9, 14]. Nonetheless, the “extrinsic noise plateau” observed in Taniguchi et al. only concerns the proteins and not the mRNAs (compare the yellow areas of Fig 6A and 6B). As a consequence, one can expect that the mechanism explaining the extrinsic noise plateau takes place at the translation level and not at the transcription. Moreover for highly expressed proteins, the protein CV is independent of the protein expression; it is therefore not gene-dependent as it would be the case for gene regulation. Finally, we have considered a simple model with gene regulation (like the three-stage model of [9]), and determined the regulations parameters in order to predict protein variance observed in [6]; we came with an activation/deactivation timescale has to be very high (several times the doubling time) in order to reproduce the variance experimentally observed, which is way above the typical biologically expected parameters.

One can also mention other possible mechanisms not represented in our models such as the fluctuations of availability of amino-acids or free RNA nucleotides in the medium, thus inducing additional fluctuations in the translation speed. Even if one can see here a clear analogy with the fluctuations in RNA-polymerase and ribosome availability (which also impact the transcription and translation speeds), the different timescales of the dynamics of amino-acids or free RNA nucleotides might result in a different effect. One can also challenge the hypothesis time intervals between events are modeled by exponentially distributed random variables: for instance, elongation times would be better represented as having Erlang distribution, that is the sum of independent exponential random variables. Yet, some results incline to say that it has a limited impact [13]. Also, in this model, the binding and initiation (either of RNA-polymerases or ribosomes) are considered as a single event. A more precise representation would be to describe them as two different processes (Reference [40] gives for instance a median transcriptional initiation time of 15t which is of the same order of magnitude as the elongation time).

One can also consider that, as this effect mainly affects proteins with the highest fluorescence, it is possible that some saturation induces a bias in the estimation of variance of highly produced proteins. To our knowledge, exhaustive measures of [6] for mean and variance of protein and mRNA concentrations have not been replicated at the same scale, so we have not been able to confront our results to other measures.

## Materials and methods

### The complete model

In this subsection is presented in detail the complete model of Fig 4 that includes the sharing of RNA-polymerases and Ribosomes (the other models being simplifications of this complete model). It represents, for any gene, both the number of mRNA and protein molecules associated with the gene inside a given cell. But, contrary to the two-stage model (Fig 1) it also explicitly represents the volume *V*(*t*) that is changing across the time *t* due to cell growth, so that, if *M*(*t*) and *P*(*t*) represent the respective number of mRNAs and proteins of a given gene at any time *t*, one can now explicitly represent their concentration by

$$\begin{array}{c} M(t)/V(t)\;\text{and}\;P(t)/V(t). \end{array}$$

Furthermore, contrary to the two-stage model, all genes in the bacteria are represented altogether here (in order to represent the global sharing of RNA-polymerases and ribosomes in the different productions of proteins) and the division (to represent a partitioning of components at septation). When dividing, the model focuses on only one of the two daughter cells in order to follow one lineage of cells.

#### Volume growth and division

The volume of the cell is represented in the model and increases alongside the growth of the cell. As the number of mRNAs and proteins of each type is represented, this volume also makes it possible to explicitly represent their concentration inside the cell. When the cell doubles its volume, division occurs: it is a sizer model. All the compounds (mRNAs and proteins) are then randomly partitioned in the two daughter cells (this partition is considered as equally likely as each compound has an equal chance to be in either one of the two daughter cells, we do not consider strong asymmetry in cell volume division). Then the model only follows one of the two daughter cells beginning a new cell cycle.

#### Units of production

Each type of protein has a specific type of mRNA and a unique gene associated with (in particular, there is no notion of operons). In the *i*-th unit of production, the number of gene copies *G*_*i*_, mRNAs *M*_*i*_, and proteins *P*_*i*_ inside the cell is explicitly represented. Each copy of the gene can be transcribed into an mRNA. The mRNA can then be translated into a protein until its degradation, the degradation rate is specific to the type of mRNA. We do not consider any rate of protein degradation: the proteolysis occurring in a timescale much longer than the cell cycle (see [17]) for most proteins, its decay is then dominated by protein partition that occurs at division.

#### DNA-replication

Each gene can be present in one or two copies in the cell (only one DNA replication is considered as in the slowly growing cells of Taniguchi et al.). The instant of replication of each gene is simply determined by its position on the chromosome. When replicated, the rate of transcription of the gene is doubled.

#### RNA-polymerases and ribosomes

The production of mRNAs and proteins respectively requires RNA-polymerases and ribosomes. The concentrations of non-allocated (or free) RNA-polymerases and ribosomes respectively determine the rates of transcriptions and translations. During the time of elongation, the RNA-polymerase (resp. ribosome) remains sequestered on the DNA (resp. the mRNA). As the cell grows, new RNA-polymerases and ribosomes are created and participate in the production of proteins.

### Analysis of each intermediate models

**Fig 7:**
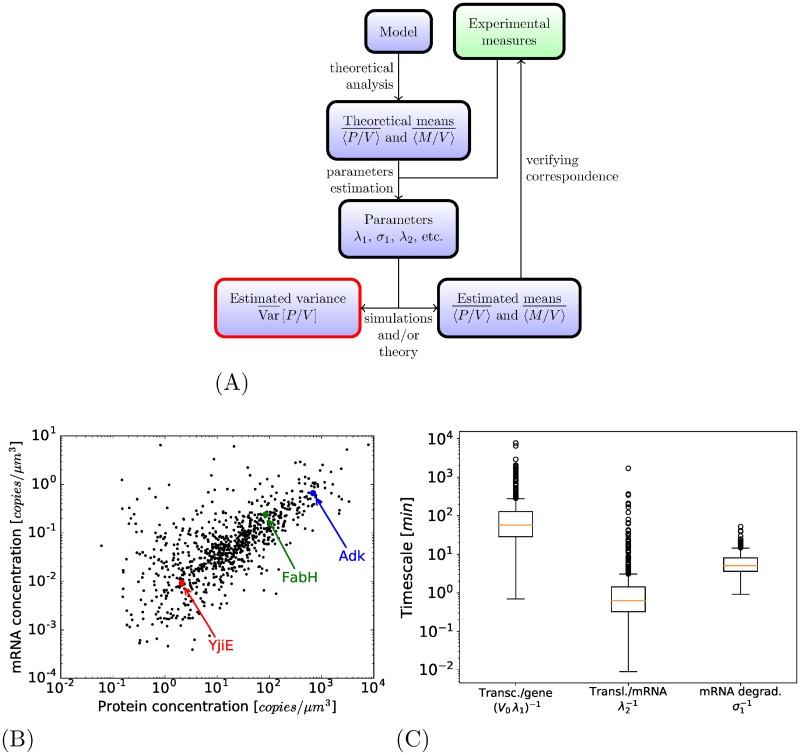
Experimental data and modeling principles. (A): The complete model. (B): Scheme of analysis to determine the variance of protein concentration for every gene predicted in each intermediate model. (C): [6] measures of mRNAs and proteins for 841 genes. (D): Box-plots presenting the parameters deduced from experiments to corresponds to the model of Fig 2; for the other models, these rates are in the same order of magnitude.

Each of the intermediate models is systematically analyzed with the same method, see Fig 7A. The average behavior of the model is analytically predicted (either with exact formulas for the first two intermediate models, or approximately for the last complete model); it makes it possible, for each of the 841 genes of Taniguchi et al. (for which both the average protein and mRNA production have been measured), to determine the set of parameters of the model. An overview of the parameters hence determined is shown in Fig 7C. Simulations are then performed—using methods derived from Gillespie algorithm—with these parameters over 10000 cell cycles: it makes it possible to check the accuracy of the average concentration and to predict the variance of the concentration of proteins. For each model, we then have the variance of protein concentration predicted for the wide range of genes measured in [6].

### Means and variances

Throughout this paper we use the notation 〈*X*(*t*)〉 and Var [*X*(*t*)] for the mean and variance of a random variable *X*(*t*) at a time *t* of the cell cycle. We introduced the concentrations for mRNAs and proteins. If *M*(*t*) and *P*(*t*) denote the random variables representing the number of mRNAs and proteins of a given type at time *t* and *V*(*t*) is the volume at this instant, the corresponding concentrations are *M*(*t*)/*V*(*t*) and *P*(*t*)/*V*(*t*). One of the goals of this work is to study the properties of the mean, 〈*P*(*t*)/*V*(*t*)〉, and variance, Var [*P*(*t*)/*V*(*t*)], of these concentrations. These quantities correspond to the mean and the variance of a population of synchronized cells of volume *V*(*t*). The measurements of [6] consider a cell population in exponential growth; by consequence, we also have to define the notions of global mean and variance for a heterogeneous population (see [33, 41, 42] for the population distribution in exponential growth). By denoting by *u* the age distribution of the cell population, we can define the global average $\bar{\langle P/V\rangle }$ and variance $\bar{\text{Var}}[P/V]$ averaged over the population,

$$\begin{array}{c} \bar{\left\langle P/V\right\rangle }=\int_{0}^{\tau_{D}}\langle P(t)/V(t)\rangle \;u\left(t\right)\;\text{d}t \end{array}$$ (6)

$$\begin{array}{c} \bar{\text{Var}}[P/V]=\int_{0}^{\tau_{D}}[\langle {\left(P(t)/V(t)\right)}^{2}\rangle -{\bar{\left\langle P/V\right\rangle }}^{2}]\;u\left(t\right)\;\text{d}t. \end{array}$$ (7)

We observe that the choice of the age distribution *u* (either uniform or corresponding to an exponentially growing population) does not seem to have much impact; see Section 2.2.3 of S1 Appendix for more details. We therefore simply considered *u* as uniform in the Results section.

### Intrinsic and extrinsic effects

As for the previous studies of [1, 12], we want to decompose protein variance that can be attributed to the intrinsic expression from the one due to extrinsic mechanisms. It appears that this decomposition can be computed using two different ways:

- First, there is the method used in Taniguchi et al. As the intrinsic noise is usually attributed to the protein production mechanism alone, a model that represents only transcription and translation, as the classic two-stage model, are usually considered as predicting the intrinsic noise.
  Yet, in our case, the consideration of a more realistic mechanism for protein disappearance (through segregation at division rather than regular decay) prevent us from using directly the two-stage models as a quantitative representation of the intrinsic variance. Our very first intermediate model, where the number of proteins and mRNAs are exactly halved at division, is considered as our baseline model. This baseline model is very close to the two-stage model in that sense that it contains no other features than those intrinsically linked to protein production. Therefore, we use protein variance predicted by this baseline model as intrinsic protein variance.
  From this baseline model, any additional variance added by the introduction to the model of external mechanisms (random partition at division, gene replication, etc.) would be considered as extrinsic. For every type of protein, we will look how the global variance $\bar{\text{Var}}[P/V]$ changes with the subsequent introduction of the external mechanisms.
- Secondly, it appears that the previous method of extrinsic noise estimation is not exactly the same from the first attempt made by [1] using the dual reporter technique. [34] showed that decomposition using the dual reporter technique can be interpreted as an estimator of the environmental state decomposition (also called the law of total variance). It decomposes protein variance between the effects specifically due to the stochastic nature of the instants of birth and death of mRNAs and proteins (intrinsic noise) and the external effect of the biological environment (extrinsic noise). If *Z* represents the state of the cell, the number of RNA-polymerases, the volume, etc…, then the protein concentration *P*/*V* can be decomposed such as,
  $$\begin{array}{c} \bar{\text{Var}}[P/V]=\underset{\text{unexplained}\;\text{by}\;Z}{\underset{︸}{\bar{{\text{Var}}_{int}}[P/V]}}+\underset{\text{explained}\;\text{by}\;Z}{\underset{︸}{\bar{{\text{Var}}_{ext}}[P/V]}}, \end{array}$$ (8)
  where:
  $$\begin{array}{cc} \bar{{\text{Var}}_{int}}[P/V] & =\int_{0}^{\tau_{D}}{\left\langle \text{Var}[P(t)/V(t)|Z(t)]\right\rangle }_{Z(t)}\;u\left(t\right)\;dt\\ \bar{{\text{Var}}_{ext}}[P/V] & =\int_{0}^{\tau_{D}}[{\left\langle {\left\langle P(t)/V(t)|Z(t)\right\rangle }^{2}\right\rangle }_{Z(t)}-{\bar{\left\langle P/V\right\rangle }}^{2}]u\left(t\right)\;dt \end{array}$$
  where ${\bar{\langle \cdot \rangle }}_{Z(t)}$ indicates the integration over the variable *Z* in a population of cells of age *t*. (Note that in the case of the two first intermediate models, the environment *Z* is simply the deterministic volume and simplified expressions for $\bar{{\text{Var}}_{int}}[P/V]$ and $\bar{{\text{Var}}_{ext}}[P/V]$ in these cases can be found in Eqs (2) and (3)).
  The variable *Z* represents the common environment in which the two similar genes of the dual reporter technique evolve; yet mathematically, it is dependent on the model that we consider (for each model, it is what is considered as being part of the “environment” of the gene). We have therefore explicitly described for each intermediate models what it represents in this context (the second intermediate model with gene replication shows an illustrative example of this decomposition). Once the variable *Z* fixed, the decomposition is explicit and separates the total variance in two parts: $\bar{{\text{Var}}_{int}}[P/V]$ that corresponds to the intrinsic contributions and $\bar{{\text{Var}}_{ext}}[P/V]$ corresponds to the variance induced by external contributions represented by the environment *Z* (volume fluctuation, concentrations of free RNA-polymerases and ribosomes, etc.) The term $\bar{{\text{Var}}_{int}}[P/V]$ is indeed the variance that can be expected from a model without any external fluctuation. A model like the two-stage model does not consider any change in the environment of protein production, so the term $\bar{{\text{Var}}_{ext}}[P/V]$ of the decomposition would be null.

In the intermediate models, we quantify the external contributions by these two means, either by looking at the increase of the global variance $\bar{\text{Var}}[P/V]$, or by performing the environmental state decomposition and looking the portion of external variance $\bar{{\text{Var}}_{int}}[P/V]/\bar{\text{Var}}[P/V]$ predicted. We have seen that these two ways to quantify the external contributions of protein variance are not strictly equivalent.

## Supporting information

S1 Appendix Additional information on models analysis. (PDF)

S1 Fig Intermediate model with volume growth and partition at division: Correspondence of simulations with experimental concentration, Fano Factor and average concentration, variance ratio and average concentration, example of dual reporter technique. (A) Comparison of the average productions of proteins (main) and mRNAs (inset) obtained in the simulations and those experimentally measured. (B) The Fano factor as a function of the average protein production: the production with the lowest Fano Factor tends to be the less expressed. (C) For each type of protein, the variance in the case of exact partition divided by the variance in the case of random partition as a function of the protein average production. (D) Example of the dual reporter technique with two promoters corresponding to the protein Adk.
(EPS)

S2 FigIntermediate model with cell cycle and gene replication: Parameters, correspondence of simulations with experimental concentration, influence of protein parameters on the the variance of its concentration, profile of a protein with an extreme low variance.(A): Quantitative summary of the parameters for this model. (B): In the case of simulations, comparison of the average productions of protein (main) and mRNAs (inset) obtained in the simulations and those experimentally measured. Note that, in this case, in addition to the simulations, we can directly use theoretical formulas to directly predict the *variance* of each protein (see Section 2 of S1 Appendix) (C): Evolution of $\bar{\text{Var}}[P/V]$ while varying successively the gene position in the DNA, the mRNA number and the mRNA lifetime while keeping $\bar{\langle P/V\rangle }$ constant. (D): Main: Profile of a modified version of AdK with higher transcription rate (approximately ten times more) and a lesser mRNA lifetime (ten times less). The variance is reduced, but it is not enough to clearly separate between the distributions at birth (at time *t* = 0) and at the replication of the gene (at time *t* = *τ*_*R*_) (Inset).(EPS)

S3 FigComplete model: Parameters, correspondence of simulations with experimental concentration, evolution of free RNA-polymerases and free ribosomes.(A): Quantitative summary of the parameters. (Different choice of $\bar{f_{Y}}$ and $\bar{f_{R}}$ when computing the parameters induce little changes for the rate of transcription per gene $\lambda_{1,i}\bar{f_{Y}}$ and the rate of translation per mRNA $\lambda_{2,i}\bar{f_{R}}$). (B): Ratio between the average concentration for protein (main figure) and mRNA (inset) in simulation and in experiments. (C) and (D): the respective means of free RNA-polymerases and ribosomes at each moment of the cell cycle in the simulations (solid lines) and the ones predicted by the system of ODEs (dashed lines). Inset: an example of the dynamics of free RNA-polymerases and ribosomes for one simulation.(EPS)

S4 Fig Three simulations of the complete model: Different levels of free ribosomes and free RNA-polymerases (1st line: Few free ribosomes, many free RNA-polymerases; 2nd line: Few free ribosomes, few free RNA-polymerases; 3rd line: Many free ribosomes, many free RNA-polymerases). (A), (C) and (E): Ratio between protein variance of the multi-protein and the gene-centered models. Inset: the histogram of these variance ratios. (B), (D) and (F): Free RNA-polymerase (above) and free ribosome (below) number distribution for cells each of the volumes. In thick lines the binomial distribution predicted for the simplified model (see Section 3.8 of S1 Appendix).
(EPS)
