# Supplementary material for: Models of protein production along the cell cycle: An investigation of possible sources of noise
Source: PLoS One. 2020 Jan 16;15(1):e0226016. doi: 10.1371/journal.pone.0226016 (PMC6964835; doi:10.1371/journal.pone.0226016)
Supplement: S1 Appendix — (PDF) [file pone.0226016.s001.pdf]

Models of protein production along the cell cycle: an investigation of possible sources of noise. - Appendix

Renaud Dessalles, Vincent Fromion*, Philippe Robert,
*vincent.fromion@inra.fr

1 Impact of Random Partitioning

Each of the three different models are analyzed following the scheme presented in Figure 7A of the main article. We begin with a theoretical analysis to derive the average production of mRNAs and proteins along the cell cycle. Using these results, we then fit the parameters of the model to make them correspond to the experimental data of [1]. Then simulations are performed to predict protein variance for each gene considered.

We present now results for the model with volume growth, constant gene concentration and partitioning at division which has been presented in Figure 2 of the main article. The results of the theoretical analysis part are similar for both cases of exact or random partitioning at division.

1.1 Theoretical Analysis

1.1.1 Messenger-RNA Dynamic

Here is shown the Proposition 1 that describes the average number of mRNAs at any instants of the cell cycle.

For any time $s \in \mathbb{R}_+$, denote by M_s the number of mRNAs at this instant. We suppose that the initial time $s = 0$ is a time of division; in this case, at each time $i \cdot \tau_D$ with $i \in \mathbb{N}$ are moments of division. For any $i \in \mathbb{N}$, $M_{i\tau_D}$ denotes the number of mRNAs at the beginning of i-th cell cycle and $M_{i\tau_D-}$ the number of mRNAs in the $(i-1)$-th cell cycle just *before* division.

We suppose that a lot of cell divisions have already occurred even before time $t = 0$, and hence the considered cell cycle takes place when the embedded Markov chain $(M_{i\tau_D})_i$ has already reached its steady state: the distribution $M_{i\tau_D}$ is the same as the distribution of $M_{(i+1)\tau_D}$. If the steady state is already reached at time 0, it implies that the distribution of any $M_{i\tau_D+t}$ for any $i \in \mathbb{N}$ and $s \in [0, \tau_D[$ is equal to the distribution of M_t. As a consequence, we can only consider the first cell cycle $t \in [0, \tau_D[$ to fully characterize the behavior of M_s at any time $s \in \mathbb{R}_+$.

We propose here to describe the evolution of (M_t) between times 0 and τ_D (during this period of time, the number of mRNA approximately doubles). We first divide mRNAs into two categories,

— First group: mRNAs which were present at the birth of the cell. Each mRNA i of the first group is characterized by $E^i_{\sigma_1}$, its lifetime given by an exponential random variable of rate σ_1. The i-th mRNA still exists at time t if and only if $E^i_{\sigma_1} > t$. As a consequence, the number of mRNAs of this group still existing at time t is given by

$$\sum_{i=1}^{M_0} \mathbf{1}_{\left\{E^i_{\sigma_1} > t\right\}}. \tag{1.1}$$

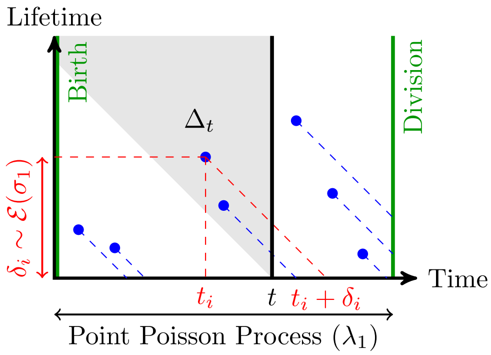

S5 Fig. Illustration of the Marked Point Poisson Process describing the dynamic of mRNAs: each mRNA is characterized by the point (x_i, y_i) (with (x_i, y_i) following the MPPP $\mathcal{N}$, whose distribution is of intensity ν). The random variable x_i represents the time at mRNA creation and y_i its lifetime, hence this mRNA exists from volume x_i up to volume $x_i + y_i$; that is to say that mRNA is still present at time t, if and only if the point (x_i, y_i) is in the set with $\Delta_t = \left\{(x, y) \in \mathbb{R}_+^2,\, 0 < x < t,\, y > t - x\right\}$.

— Second group: mRNAs which have been created since the birth of the cell. The description of the number of mRNA of this group is more complicated. It is necessary to resort to the framework of Marked Poisson Point Processes (MPPP). An MPPP is a two-dimensional process. It is based on a Poisson process where each of its random point is "marked" with another random variable; each point of a MPPP is a couple (x, y) where x is part of a Poisson point process and y is the mark distributed according to a certain distribution. One can refer to the first Chapter of [2] or [3] for the main results concerning MPPP.

We use this tool to characterize the number of mRNAs of the second group. In our case, the first variable x represents the time at which the mRNA is created and the second variable y represents the mRNA lifetime. Define $\mathcal{N}$ an MPPP of intensity

$$\nu(\mathrm{d}x, \mathrm{d}y) = \lambda_1 V(x)\,\mathrm{d}x \otimes \sigma_1 e^{-\sigma_1 y}\,\mathrm{d}y.$$

It is noticeable that the underlying Poisson Process of this MPPP is not homogeneous. If the i-th mRNA of this group is born at time x_i and its lifetime is y_i, then it exists at time t if and only if $(x_i, y_i) \in \Delta_t$ with

$$\Delta_t = \left\{(x, y) \in \mathbb{R}_+^2,\, 0 < x < t,\, y > t - x\right\}.$$

One can refer to S5 Fig. Therefore the number of mRNAs of this group still present at time t is given by

$$\mathcal{N}(\Delta_t) = \iint_{\mathbb{R}_+^2} \mathbf{1}_{\{(x,y)\in\Delta_t\}} \mathcal{N}(\mathrm{d}x, \mathrm{d}y)\,. \tag{1.2}$$

By summing the number of mRNAs for each group (Equations (1.1) and (1.2)), it follows the total number of mRNAs present at time $t \in [0, \tau_D[$:

$$M_t = \sum_{i=1}^{M_0} \mathbf{1}_{\left\{E_{\sigma_1}^i > t\right\}} + \mathcal{N}(\Delta_t)\,. \tag{1.3}$$

This description of the dynamic of M_t, together with the steady state hypothesis which implies that $M_0 \overset{\mathcal{D}}{=} M_{\tau_D}$, allows to prove the next proposition.

Proposition 1. *At steady state, the concentration of mRNAs at time $t \in [0, \tau_D[$ of the cell cycle is*

$$\langle M_t/V(t)\rangle = \frac{\lambda_1 \tau_D}{\sigma_1 \tau_D + \log 2}.$$

Proof. By taking the mean of Equation (1.3), it follows for any time t of the cell cycle:

$$\langle M_t \rangle = \left\langle \sum_{i=1}^{M_0} \mathbf{1}_{\left\{E^i_{\sigma_1} > t\right\}} \right\rangle + \langle \mathcal{N}\left(\Delta_t\right)\rangle .$$

Since all $\left(E^i_{\sigma_1}\right)_i$ are i.i.d. and independent of M_0, the first term is given by

$$\left\langle \sum_{i=1}^{M_0} \mathbf{1}_{\left\{E^i_{\sigma_1} > t\right\}} \right\rangle = \langle M_0 \rangle e^{-t\sigma_1}.$$

For the second term, one has to remark that as $\mathcal{N}$ is a MPPP, $\mathcal{N}\left(\Delta_{\tau_D -}\right)$ is a Poisson random variable (Proposition 1.13.a of [2]). The parameter of this Poisson random variable is given by

$$\nu\left(\Delta_t\right) = \iint_{\Delta_t} \nu\left(\mathrm{d}x, \mathrm{d}y\right) = V_0 \frac{\lambda_1 \sigma_1}{\log 2 + \sigma_1 \tau_D}\left(2^{t/\tau_D} - e^{-\sigma_1 t}\right).$$

As a consequence, one gets that, for any time t in the cell cycle,

$$\langle M_t \rangle = \langle M_0 \rangle e^{-t\sigma_1} + V_0 \frac{\lambda_1 \tau_D}{\tau_D \sigma_1 + \log 2} \cdot \left(2^{t/\tau_D} - e^{-t\sigma_1}\right).$$

We still have to specify the mean number of mRNAs at birth $\langle M_0 \rangle$. At the end of the cell cycle, for $t = \tau_D -$, the average number of mRNAs is given by

$$\langle M_{\tau_D -} \rangle = \langle M_0 \rangle e^{-\tau_D \sigma_1} + V_0 \frac{\lambda_1 \tau_D}{\tau_D \sigma_1 + \log 2} \cdot \left(2 - e^{-\tau_D \sigma_1}\right),$$

and since at steady state,

$$\langle M_{\tau_D} \rangle = \langle M_0 \rangle = \langle M_{\tau_D -} \rangle / 2.$$

Hence

$$\langle M_0 \rangle \left(2 - e^{-\tau_D \sigma_1}\right) = V_0 \frac{\lambda_1 \tau_D}{\tau_D \sigma_1 + \log 2} \cdot \left(2 - e^{-\tau_D \sigma_1}\right),$$

which gives the result. □

In particular, as the mean $\langle M_t/V(t)\rangle$ does not change across the cell cycle, the global mRNA average $\overline{\langle M/V \rangle}$ (as it is defined in Equation (6) of the main article), does not depend on the choice of the population distributions u in this case. It is given by,

$$\overline{\langle M/V \rangle} = \frac{\lambda_1 \tau_D}{\sigma_1 \tau_D + \log 2}. \tag{1.4}$$

1.1.2 Protein Dynamic

The mean number of mRNAs is now determined for any moment of the cell cycle. Each of the mRNAs potentially produces proteins at rate λ_2. As for the mRNAs, we describe the number of proteins at time t by grouping them into two categories.

— The P_0 proteins that were present at birth and which remain in the bacteria during all the cell cycle (as said in the main article the proteolysis is not considered in this model).

— The proteins that have been created during the current cell cycle. The rate of production is depending on the current number of mRNAs. We consider $\mathcal{N}^i_{\lambda_2}$ (for $i \in \mathbb{N}$ and $i \geq 1$) independent Poisson Point Processes of intensity λ_2. If the i-th mRNA exists at time t (that is to say if $i \leq M_t$), then the number of proteins produced by this mRNA between t and $t + \mathrm{d}t$ is $\mathcal{N}^i_{\lambda_2}(\mathrm{d}t)$.

To sum up, the number of proteins at a time t of the cell cycle is given by

$$P_t = P_0 + \sum_{i=1}^{\infty} \int_0^t \mathbf{1}_{\{i \leq M_u\}} \mathcal{N}^i_{\lambda_2}(\mathrm{d}u) \,. \tag{1.5}$$

The first term is the number of proteins at birth, and the second take into account all the proteins created between times 0 and t. One can then determine the mean number of proteins at any time t of the cell cycle.

Proposition 2. *At steady state, the concentration of proteins at any time $t \in [0, \tau_D[$ of the cell cycle is*

$$\langle P_t / V(t) \rangle = \frac{\lambda_2 \tau_D}{\log 2} \cdot \frac{\lambda_1 \tau_D}{\sigma_1 \tau_D + \log 2}.$$

Proof. By taking the average of Equation (1.5), one gets

$$\begin{aligned} \langle P_t \rangle = \langle P_0 \rangle + \sum_{i=1}^{\infty} \left\langle \int_0^t \mathbf{1}_{\{i \leq M_u\}} \mathcal{N}^i_{\lambda_2}(\mathrm{d}u) \right\rangle &= \langle P_0 \rangle + \sum_{i=1}^{\infty} \left\langle \int_0^t \mathbf{1}_{\{i \leq M_u\}} \lambda_2 \, \mathrm{d}u \right\rangle \\ &= \langle P_0 \rangle + \lambda_2 \int_0^t \left\langle \sum_{i=1}^{\infty} \mathbf{1}_{\{i \leq M_u\}} \right\rangle \mathrm{d}u = \langle P_0 \rangle + \lambda_2 \int_0^t \langle M_u \rangle \, \mathrm{d}u. \end{aligned}$$

As we know the mean number of mRNAs $\langle M_u \rangle$ at time u of the cell cycle with Proposition 1,

$$\langle P_t \rangle = \langle P_0 \rangle + \frac{\lambda_2 \tau_D}{\log 2} \cdot \frac{\lambda_1 \tau_D}{\sigma_1 \tau_D + \log 2} \cdot (V(t) - V_0) \,.$$

Since the system is at steady state, we have for time τ_D-, $\langle P_{\tau_D-} \rangle = 2 \langle P_0 \rangle$; so

$$\langle P_0 \rangle = \frac{\lambda_2 \tau_D}{\log 2} \cdot \frac{\lambda_1 \tau_D}{\sigma_1 \tau_D + \log 2} \cdot (V(\tau_D-) - V_0) = \frac{\lambda_2 \tau_D}{\log 2} \cdot \frac{\lambda_1 \tau_D}{\sigma_1 \tau_D + \log 2} \cdot V_0.$$

Consequently, for any time t of the cell cycle,

$$\langle P_t \rangle = \lambda_2 \frac{\tau_D}{\log 2} \cdot \frac{\lambda_1 \tau_D}{\sigma_1 \tau_D + \log 2} \cdot V_0 \left(1 + 2^{t/\tau_D} - 1\right);$$

hence the result. □

In particular, as the mean $\langle P_t / V(t) \rangle$ does not change across the cell cycle, the global protein average is given by,

$$\overline{\langle P/V \rangle} = \frac{\lambda_2 \tau_D}{\log 2} \cdot \frac{\lambda_1 \tau_D}{\sigma_1 \tau_D + \log 2}. \tag{1.6}$$

1.2 Parameters Estimation

For each gene measured in [1], we want to identify the set of corresponding parameters λ_1, σ_1 and λ_2. We also need to determine the "global" quantities τ_D and V_0. We first determine the parameters common to all genes. The division time τ_D is set to 150min in the article and the volume at birth V_0 is taken equal to $1.3\,\mu\text{m}^3$.[1]

Then we have to determine for each gene the three gene-specific parameters λ_1, σ_1 and λ_2. We consider the genes of the article for which was measured the empirical mean of messengers μ_m and proteins μ_p concentrations, as well as the mRNA half-life time τ_m.

First we determine the rate of mRNA degradation for each gene with the measured mRNA half-life time τ_m. A half-life τ_m indicates that a mRNA has a probability 1/2 to disappear within a duration τ_m, hence $e^{-\sigma_1 \tau_m} = 1/2$. From that, we can compute the rate σ_2 (specific for each type of mRNA),

$$\sigma_1 = \log 2/\tau_m.$$

Then we can identify the averages of mRNA and protein concentrations of the model (respectively $\overline{\langle M/V \rangle}$ and $\overline{\langle P/V \rangle}$) with the empirical averages μ_m of mRNA concentration and μ_p of protein concentration of the article. With Equations (1.4) and (1.6), the parameters λ_1 and λ_2 are

$$\lambda_1 = \mu_m \cdot \frac{\sigma_1 \tau_D + \log 2}{\tau_D}, \qquad \lambda_2 = \mu_p \cdot \frac{\log 2}{\tau_D} \cdot \frac{\sigma_1 \tau_D + \log 2}{\lambda_1 \tau_D}.$$

A summary of the different parameters can be seen in Figure 7C of the main article. Having determined all the parameters allows to perform simulations of the model using stochastic algorithm in order to assess the variability of every protein and compare them with those experimentally obtained in [1].

1.3 Simulations

When performing simulations, one needs to take care of the non-homogeneity of the Poisson processes describing mRNA creation times: the rate of protein production $\lambda_1 V(t)$ is not a homogeneous rate as it changes with time. That does not allow a direct application of Gillepsie method [4], an extension for non-homogenous processes has to be used.

Reference [4] describes an algorithm to simulate stochastic trajectories such as the quantities of different chemical species interacting together. The main idea is to consider the state of a system (for instance the number of each chemical compounds) and to compute the first reaction to occur, as well as the time when it happens. Once both pieces of information computed, one change the current state of the system accordingly with the reaction, and update the time.

One important hypothesis is that all reactions occur at exponential times (even if the rates of these exponential times may depend on the current state of the system). In the current intermediate model, at any time t, the state is described by (M_t, P_t) (respectively, the number of mRNAs and proteins), and the rate of mRNA production is $\Lambda(t) = \lambda_1 V(t)$ with λ_1 a parameter and $V(t)$ the non-constant volume of the cell. The parameter $\Lambda(t)$ does not depend on the state (M_t, P_t) but is time dependent through $V(t)$; for this reason, it is not an exponential time.

In this case, the duration of time T until the next mRNA production is characterized by

$$\mathbb{P}\left[T > x\right] = \exp\left(-\int_0^x \Lambda(t)\ \mathrm{d}t\right), \quad x > 0.$$

[1] The value of V_0, even if it is not explicitly given in [1] can be deduced from the typical width given in its supplementary materials.

which is not an exponential distribution as Λ is non-constant. To compute T, we consider that $\Lambda(t)$ is strictly positive for any $t \in \mathbb{R}_+$, as a consequence $F(x) := \int_0^x \Lambda(t)\ \mathrm{d}t$ is strictly increasing. Let E be an exponential random variable with parameter 1. We have hence

$$\forall y > 0 \quad \mathbb{P}\left[E > y\right] = \exp\left(-y\right).$$

If we consider the case of $y = F(x)$, since F is strictly increasing, hence

$$\mathbb{P}\left[E > y\right] = \exp\left(-F(x)\right) \qquad \text{and} \qquad \mathbb{P}\left[E > y\right] = \mathbb{P}\left[F^{-1}(E) > x\right].$$

As a consequence the random variable $F^{-1}(E)$ has the same distribution as T.

Based on that we can propose a new version of the algorithm of Gillespie that can take into account non-exponential times such as T.

Algorithm 1. *The equivalent of Gillespie algorithm that considers non-homogeneous events is*

1. *Initialization: Initialize time of molecules in the system and the time.*
2. *Next exponential event: determine the next event that occurs at an exponential time as in Gillespie algorithm.*
3. *Next non-homogeneous event: determine the next event that occurs at non-homogeneous rates with the method previously described.*
4. *Update: choose between events of Step 2 or Step 3 that happen first. Update the time and the molecule count accordingly.*
5. *Iterate: Consider again the Step 2 unless it has reached the end of the simulation.*

1.4 Environmental State Decomposition

As explained in the main article, the dual reporter technique [5] that compares the expression of two similar genes (with the same promoter and RBS, at an equivalent position on the chromosome) in the same cell can be interpreted as an estimator of the environmental state decomposition [6]. If Z the cell state (i.e., the common environment in which the two genes of the dual reporter technique are expressed), then we can apply the law of total variance on the protein number,

$$\overline{\mathrm{Var}}\left[P/V\right] = \underbrace{\overline{\left\langle \overline{\mathrm{Var}}\left[P/V|Z\right]\right\rangle}}_{\overline{\mathrm{Var}}_{int}[P/V]} + \underbrace{\overline{\mathrm{Var}}\left[\overline{\langle P/V|Z\rangle}\right]}_{\overline{\mathrm{Var}}_{ext}[P/V]}.$$

In order to be applied here, we need to specify what does the gene environment Z refers to. In our model, two similar genes (with the same parameters λ_1, σ_1,λ_2) in the same cell would undergo the same volume growth. But each gene would undergo a specific partition at division (for instance, in the case of random partitioning, the partitions of the proteins of one of the gene, is uncorrelated with the partition of the proteins of the other gene).

With only considering the volume as the gene environment, we end up with the following decomposition,

$$\overline{\mathrm{Var}_{int}}\left[P/V\right] = \frac{1}{\tau_D}\int_0^{\tau_D} \mathrm{Var}\left[P_t/V(t)\right]\mathrm{d}t,$$
$$\overline{\mathrm{Var}_{ext}}\left[P/V\right] = \frac{1}{\tau_D}\int_0^{\tau_D} \langle P_t/V(t)\rangle^2\,\mathrm{d}t - \overline{\langle P/V\rangle}^2.$$

As in this model, the $\langle P_t/V(t)\rangle$ remains constant during the cell cycle, the second term of the decomposition remains null.

It has been confirmed by a simulation of the dual reporter technique, where the expression of two identical promoters in the same cell has been compared (we took the example of the protein Adk). The concentrations of the two proteins, respectively P_1/V and P_2/V, have been compared. In particular, we measured their covariance which is much smaller than their respective variances (see S1 Fig (D)).

1.5 Simplified model for the random partitioning

Here we explain the simplified model used to make the prediction of protein noise ratio in blue dash line of Figure 2D of the main article.

For a given quantity P associated with the gene, the partitioning can be performed in two ways, either exact or random. The result in each case will be denoted respectively by P_e and P_r. During division, the volume is divided by two, changing from $2V_0$ to V_0. In order to be plotted in Figure 2D of the main article, we need to consider the variance of protein concentration after division

$$\eta := \frac{\mathrm{Var}\left[P_r/V_0\right]}{\mathrm{Var}\left[P_e/V_0\right]} \quad \text{as a function of} \quad x := \frac{\mathrm{Var}\left[P/(2V_0)\right]}{\langle P/(2V_0)\rangle}.$$

Proposition 3. *The Variance ratio η as a function of x is given by*

$$\eta = \frac{2V_0 x + 1}{2V_0 x}.$$

Proof. We have the quantity before division P. Since by definition, we have that $P_e = P/2$ and

$$\langle P_e/V_0\rangle = \langle P/(2V_0)\rangle\,, \quad \mathrm{Var}\left[P_e/V_0\right] = \mathrm{Var}\left[P/(2V_0)\right].$$

For the effect of binomial division, see Lemma 11, it describes the effect of the binomial division on the means and on the variances of several quantities. By the volume in order to observe the concentrations, one gets that

$$\langle P_r/V_0\rangle = \frac{\langle P\rangle}{2V_0} = \langle P/\left(2V_0\right)\rangle$$

and

$$\mathrm{Var}\left[P_r/V_0\right] = \frac{\mathrm{Var}\left[P_r\right]}{V_0^2} = \frac{\mathrm{Var}\left[P\right] + 2\langle P_r\rangle}{4V_0^2} = \mathrm{Var}\left[P/\left(2V_0\right)\right] + \frac{\langle P/(2V_0)\rangle}{2V_0}.$$

As a consequence, this gives the relation

$$\eta = \frac{\mathrm{Var}\left[P/\left(2V_0\right)\right] + \langle P/(2V_0)\rangle/(2V_0)}{\mathrm{Var}\left[P/(2V_0)\right]} = \frac{2V_0 x + 1}{2V_0 x}$$

□

2 Impact of Gene Replication

We present here results relative to the model presented in Figure 3 of the main article with volume growth, partition at division and gene replication. For this intermediate model, we still follow the analysis scheme presented in Figure 7A of the main article, but here, we are also able to produce analytic results for the variance of mRNAs and proteins.

S6 Fig. The illustration of the Marked Point Poisson Processes that describe the dynamic of mRNAs: each mRNA is characterized by the point (t_i, δ_i), with (t_i, δ_i) following MPPP $\mathcal{N}$ or $\mathcal{N}'$. Both MPPPs are of intensity ν. The random variable t_i represents its birth time and δ_i its lifetime, hence this mRNA exists from time t_i up to time $t_i + \delta_i$. The only difference for the two processes is the starting time: the process $\mathcal{N}$ in (A) begins at birth (in particular, an mRNA is still present at time t, if and only if the point (t_i, δ_i) is in the set $\Delta_t = \{(x, y),\, 0 < x < t,\, y > t - x\}$); the process $\mathcal{N}'$, in (B), begins at replication (in particular an mRNA is still present at time t, if and only if the point (t_i, δ_i) is in the set $\Delta'_t = \{(x, y),\, \tau_R < x < t,\, y > t - x\}$).

2.1 Theoretical analysis

2.1.1 Dynamics of mRNA number

The aim of this section is to prove Theorem 5 which states that at any time of the cell, the mRNA number follows a Poisson distribution. To do so, we first give a description of the number mRNAs at any time in the cell cycle using a Marked Poisson Point Process. With this description, we will be able to show Proposition 4, that the distribution of M_0 at the beginning of the cell cycle is a Poisson distribution. This proposition will allow to finally prove the main theorem of the subsection.

If time $t = 0$ is the beginning of a new cell cycle and if the system is already at steady state in the same sense as the previous models (see Section 1.1.1). We consider that M_0, the number of mRNAs at birth is known. As in Section 1.1.1, we assort mRNAs in independent groups; here we consider three categories.

— mRNAs which were present at the birth of the cell. Each of them is characterized by its lifetime given by an exponential time of rate σ_1. The i-th mRNA is still present at time t if and only if $E^i_{\sigma_1} > t$, with $\left(E^i_{\sigma_1}\right)$ being i.i.d. exponential random variables of parameter σ_1.

— mRNAs created since the birth of the cell by the first copy of the gene. The i-th mRNA of this group is characterized by the time of creation t_i given by a Poisson Process of rate λ_1 and its lifetime δ_i given by an exponential time of rate σ_1.

— mRNAs created since the gene replication by the second copy of the gene. As in the previous group, the i-th mRNA is characterized by the time of creation t_i given by a Poisson Process of rate λ_1 and its lifetime δ_i given by an exponential time of rate σ_1. But here, the Poisson Process of rate λ_1 begins at time τ_R, the time of replication of the gene.

As in Section 1.1.1, one can represent the number of mRNAs of the second and the third group as two independent MPPPs $\mathcal{N}$ and $\mathcal{N}'$. The first variable x of each of these MPPPs will represent the time. The intensity of each of the MPPP is the same,

$$\nu(\mathrm{d}x, \mathrm{d}y) = \lambda_1 \,\mathrm{d}x \otimes \sigma_1 e^{-\sigma_1 y} \,\mathrm{d}y.$$

The only difference between $\mathcal{N}$ and $\mathcal{N}'$ is the fact that they begin at time 0 for $\mathcal{N}$ and at time τ_R for $\mathcal{N}'$ (see S6 Fig). As a consequence, if we consider an mRNA of either group, the conditions of its existence at time $t \in [0, \tau_D[$ are respectively,

— if it is in the second group: $(t_i, \delta_i) \in \Delta_t$ with $\Delta_t = \{(x, y),\, 0 < x < t,\, y > t - x\}$,

— if it is in the third group: $(t_i, \delta_i) \in \Delta'_t$ with $\Delta'_t = \{(x, y),\, \tau_R < x < t,\, y > t - x\}$.

Hence, we can describe the number of mRNAs at any time $t \in [0, \tau_D[$ as follows,

$$M_t = \sum_{i=1}^{M_0} \mathbf{1}_{\left\{E^i_{\sigma_1} > t\right\}} + \mathcal{N}\left(\Delta_t\right) + \mathbf{1}_{\{t \geq \tau_R\}} \mathcal{N}'_{\lambda_1}\left(\Delta'_t\right). \tag{2.1}$$

Each term corresponds to each group of mRNAs previously described.

At first we want to characterize the distribution of M_0, the number of mRNAs at the birth of the cell. To do so, we use the steady state hypothesis that implies that $M_0 \stackrel{\mathcal{D}}{=} M_{\tau_D}$.

Proposition 4. *At steady state, the number of mRNAs at birth M_0 follows a Poisson distribution of parameter:*

$$x_0 = \frac{\lambda_1}{\sigma_1}\left[1 - \frac{e^{-(\tau_D - \tau_R)\sigma_1}}{2 - e^{-\tau_D \sigma_1}}\right].$$

Proof. When $s{=}\tau_D-$, by Relation (2.1),

$$M_{\tau_D -} = \sum_{i=1}^{M_0} \mathbf{1}_{\left\{E^i_{\sigma_1} > \tau_D -\right\}} + \mathcal{N}_{\lambda_1}\left(\Delta_{\tau_D -}\right) + \mathcal{N}'_{\lambda_1}\left(\Delta'_{\tau_D -}\right).$$

The first term corresponds to initial messengers not degraded after the time τ_D. Suppose that M_0 is distributed according to a Poisson distribution with parameter x_0, then the random variable

$$\sum_{i=1}^{M_0} \mathbf{1}_{\left\{E^i_{\sigma_1} > \tau_D -\right\}}$$

follows also a Poisson distribution with parameter $x_0 e^{-\tau_D \sigma_1}$. Operation of thinning of Poisson processes, see [3] for example.

The second term corresponds to mRNAs that were created by the first copy of the gene and which are still present at division. Since $\mathcal{N}$ is a MPPP, $\mathcal{N}\left(\Delta_{\tau_D -}\right)$ is a Poisson random variable (Proposition 1.13 of [2]) with parameter

$$\nu\left(\Delta_{\tau_D -}\right) = \int_0^{\tau_D} \int_{\tau_D - x}^{\infty} \lambda_1 \sigma_1 e^{-\sigma_1 y} \, \mathrm{d}y \, \mathrm{d}x = \frac{\lambda_1}{\sigma_1}\left(1 - e^{-\tau_D \sigma_1}\right).$$

The third term corresponds to mRNAs that were created by the second copy of the gene (replicated at time τ_R) and which are still present at division. As before, $\mathcal{N}'\left(\Delta'_{\tau_D -}\right)$ is a Poisson random variable with parameter

$$\nu\left(\Delta'_{\tau_D -}\right) = \int_{\tau_R}^{\tau_D} \int_{\tau_D - x}^{\infty} \lambda_1 \sigma_1 e^{-\sigma_1 y} \, \mathrm{d}y \, \mathrm{d}x = \frac{\lambda_1}{\sigma_1}\left(1 - e^{-(\tau_D - \tau_R)\sigma_1}\right).$$

As $M_{\tau_D -}$ is the sum of three independent Poisson random variables, one gets that

$$\begin{aligned} M_{\tau_D -} &\sim \mathcal{P}\left(x_0 e^{-\sigma_1 \tau_D} + \frac{\lambda_1}{\sigma_1}\left(1 - e^{-\tau_D \sigma_1}\right) + \frac{\lambda_1}{\sigma_1}\left(1 - e^{-(\tau_D - \tau_R)\sigma_1}\right)\right) \\ &\sim \mathcal{P}\left(x_0 e^{-\sigma_1 \tau_D} + \frac{\lambda_1}{\sigma_1}\left(2 - e^{-\tau_D \sigma_1} - e^{-(\tau_D - \tau_R)\sigma_1}\right)\right). \end{aligned}$$

Between τ_D- and τ_D, with the random sampling, each mRNA has an equal chance to stay or to disappears, therefore

$$M_{\tau_D} = \sum_{1=0}^{M_{\tau_D -}} B_{1/2,i}$$

with $\left(B_{1/2,i}\right)$ i.i.d. Bernoulli random variables with parameter 1/2. The random variable $B_{1/2,i}$ determines if the i-th mRNA is in the next considered cell or not. The random variable M_{τ_D} hence follows a Poisson distribution such that

$$M_{\tau_D} \sim \mathcal{P}\left(\left[x_0 e^{-\sigma_1\tau_D} + \frac{\lambda_1}{\sigma_1}\left(2 - e^{-\tau_D\sigma_1} - e^{-(\tau_D-\tau_R)\sigma_1}\right)\right]\Big/ 2\right).$$

Since the system is at steady state, one has $M_0 \overset{\mathcal{D}}{=} M_{\tau_D}$, therefore

$$x_0 = \frac{1}{2}\left(x_0 e^{-\sigma_1\tau_D} + \frac{\lambda_1}{\sigma_1}\left(2 - e^{-\tau_D\sigma_1} - e^{-(\tau_D-\tau_R)\sigma_1}\right)\right),$$

which gives

$$x_0 = \frac{\lambda_1}{\sigma_1}\left[1 - \frac{e^{-(\tau_D-\tau_R)\sigma_1}}{2 - e^{-\tau_D\sigma_1}}\right].$$

Since the steady state distribution is unique, the number of mRNAs at birth follows a Poisson distribution of parameter x_0 at steady state. □

We have determined the steady state distribution of the embedded Markov Chain $(M_{i\tau_D})_{i\in\mathbb{N}}$. Now, we analyze the distribution of mRNA number at any instant t of the cell cycle.

Theorem 5. *At steady state, at time t in the cell cycle, the distribution of the mRNA number M_t is Poisson with parameter*

$$x_t = \frac{\lambda_1}{\sigma_1}\left[1 - \frac{e^{-(t+\tau_D-\tau_R)\sigma_1}}{2 - e^{-\tau_D\sigma_1}} + \mathbf{1}_{\{t\geq\tau_R\}}\left(1 - e^{-(t-\tau_R)\sigma_1}\right)\right].$$

In particular, the mean and the variance of mRNA concentration are known at any time t of the cell cycle,

$$\langle M_t/V(t)\rangle = \frac{\lambda_1}{\sigma_1 V(t)}\left[1 - \frac{e^{-(t+\tau_D-\tau_R)\sigma_1}}{2 - e^{-\tau_D\sigma_1}} + \mathbf{1}_{\{t\geq\tau_R\}}\left(1 - e^{-(t-\tau_R)\sigma_1}\right)\right],$$

$$\mathrm{Var}\left[M_t/V(t)\right] = \frac{\lambda_1}{\sigma_1 V(t)^2}\left[1 - \frac{e^{-(t+\tau_D-\tau_R)\sigma_1}}{2 - e^{-\tau_D\sigma_1}} + \mathbf{1}_{\{t\geq\tau_R\}}\left(1 - e^{-(t-\tau_R)\sigma_1}\right)\right].$$

Proof. At a moment t of the cell cycle, the moment-generating function of M_t at $\xi < 0$ is given by

$$\langle \exp(\xi M_t)\rangle = \left\langle \exp\left(\xi\left(\sum_{i=1}^{M_0}\mathbf{1}_{\left\{E^i_{\sigma_1}>t\right\}} + \mathcal{N}(\Delta_t) + \mathbf{1}_{\{t\geq\tau_R\}}\mathcal{N}'_{\lambda_1}(\Delta'_t)\right)\right)\right\rangle.$$

Since M_0, $E^i_{\sigma_1}$, $\mathcal{N}_{\lambda_1}$ and $\mathcal{N}'_{\lambda_1}$ are all independent, it follows that

$$\langle \exp(\xi M_t)\rangle = \left\langle \exp\left(\sum_{i=0}^{M_0}\xi\mathbf{1}_{\left\{E^i_{\sigma_1}>t\right\}}\right)\right\rangle \cdot \langle \exp(\xi\mathcal{N}(\Delta_t))\rangle \cdot \left\langle \exp\left(\xi\mathbf{1}_{\{t\geq\tau_R\}}\mathcal{N}'(\Delta'_t)\right)\right\rangle.$$

For the first factor, since all the random variables $\mathbf{1}_{\left\{E^i_{\sigma_1}>t\right\}}$ are i.i.d. Bernoulli variables with parameter $e^{-t\sigma_1}$ and independent of M_0, one has

$$\begin{aligned}\left\langle \exp\left(\sum_{i=0}^{M_0}\xi\mathbf{1}_{\left\{E^i_{\sigma_1}>t\right\}}\right)\right\rangle &= \left\langle\left\langle \exp\left(\xi\mathbf{1}_{\left\{E^1_{\sigma_1}>t\right\}}\right)|M_0\right\rangle^{M_0}\right\rangle \\ &= \left\langle \exp\left(1 + e^{-t\sigma_1}(e^{\xi}-1)\right)^{M_0}\right\rangle.\end{aligned}$$

With Proposition 4, M_0 is known to be a Poisson random variable of parameter x_0, hence, with the probability generating function of a Poisson random variable,

$$\left\langle \exp\left(\sum_{i=0}^{M_0} \xi \mathbf{1}_{\{E^i_{\sigma_1} > t\}}\right)\right\rangle = \left\langle \exp\left(x_0 e^{-t\sigma_1}\left(e^{\xi}-1\right)\right)\right\rangle$$

holds. For the second factor, one can recall that $\mathcal{N}(\Delta_t)$ is a Poisson random variable. As in Proposition 4, its parameter can be calculated

$$\nu(\Delta_t) = \int_0^t \int_{\tau_D - x}^{\infty} \lambda_1 \sigma_1 e^{-\sigma_1 y}\, \mathrm{d}y\, \mathrm{d}x = \frac{\lambda_1}{\sigma_1}\left(1 - e^{-t\sigma_1}\right).$$

Identically for the third factor, $\mathcal{N}'(\Delta'_t)$ is a Poisson random variable of parameter

$$\nu(\Delta'_t) = \int_{\tau_R}^t \int_{\tau_D - x}^{\infty} \lambda_1 \sigma_1 e^{-\sigma_1 y}\, \mathrm{d}y\, \mathrm{d}x = \frac{\lambda_1}{\sigma_1}\left(1 - e^{-(t-\tau_R)\sigma_1}\right).$$

As a consequence, the moment generating function of M_t is

$$\begin{aligned}\left\langle \exp(\xi M_t)\right\rangle &= \left\langle \left(x_0 e^{-t\sigma_1} + \frac{\lambda_1}{\sigma_1}\left(1-e^{-t\sigma_1}\right) + \mathbf{1}_{\{t>\tau_R\}}\frac{\lambda_1}{\sigma_1}\left(1-e^{-(t-\tau_R)\sigma_1}\right)\right)\left(e^{\xi}-1\right)\right\rangle \\ &= \left\langle \left(x_0 e^{-t\sigma_1} + \frac{\lambda_1}{\sigma_1}\left(1-e^{-t\sigma_1} + \mathbf{1}_{\{t>\tau_R\}}\left(1-e^{-(t-\tau_R)\sigma_1}\right)\right)\right)\left(e^{\xi}-1\right)\right\rangle\end{aligned}$$

which is the moment-generating function of a Poisson random variable of parameter

$$x_0 e^{-t\sigma_1} + \frac{\lambda_1}{\sigma_1}\left(1-e^{-t\sigma_1} + \mathbf{1}_{\{t>\tau_R\}}\left(1-e^{-(t-\tau_R)\sigma_1}\right)\right).$$

□

2.1.2 Dynamics of Proteins

As for the previous analysis of the mRNA number, we search an expression for protein production through the cell cycle. This case is more complicated than the mRNA case and we will only calculate analytical expressions only for the first two moments of P_t.

Propositions 8 and 10 are the main theoretical results of this section: for any time t of the cell cycle, it gives explicit expressions for the mean $\langle P_t\rangle$ and the variance $\mathrm{Var}[P_t]$ of the protein number. This result is important as it will be used to directly calculate the mean $\overline{\langle P/V\rangle}$ and variance $\overline{\mathrm{Var}}[P/V]$ of the protein concentration averaged across the cell cycle without using simulations: only with the parameters of the model (λ_1, σ_1, λ_2, τ_R and τ_D), we will be able to know the behavior of the protein concentration in terms of variance.

In order to prove the Propositions 8 and 10, we will characterize $\langle P_t\rangle$ and $\mathrm{Var}[P_t]$ in the two following cases:

1. First, we consider the case before replication ($t < \tau_R$). We begin by considering that the state of the cell at birth (M_0, P_0) is known and we calculate the first two moments of P_t for any time $t < \tau_R$ (Corollary 7). Then, we integrate over all the possible initial states (M_0, P_0) to determine expressions for $\langle P_t\rangle$ and $\mathrm{Var}[P_t]$ for any time $t < \tau_R$ (Proposition 8). These expressions are dependent of the first moments of (M_0, P_0): they depend on $\langle M_0\rangle$, $\langle P_0\rangle$, $\mathrm{Var}[M_0]$, $\mathrm{Var}[P_0]$ and $\mathrm{Cov}[M_0, P_0]$.

2. Then we consider the case after replication ($t \geq \tau_R$). Similarly the first case, we will consider that the state of the cell at replication (M_{τ_R}, P_{τ_R}) is known and we calculate the first two moments of P_t for any time $\tau_R \leq t < \tau_D$ (Proposition 10). After integration, expressions for $\langle P_t \rangle$ and $\mathrm{Var}[P_t]$ for any time t after replication are determined, these expressions depend on $\langle M_{\tau_R} \rangle$, $\langle P_{\tau_R} \rangle$, $\mathrm{Var}[M_{\tau_R}]$, $\mathrm{Var}[P_{\tau_R}]$ and $\mathrm{Cov}[M_{\tau_R}, P_{\tau_R}]$ (Proposition 10).

In the end, in Propositions 8 and 10, are presented the mean and variance of protein number at any time t of the cell cycle, only depending on the first moments of (M_0, P_0) and (M_{τ_R}, P_{τ_R}). Additional results then determine explicitly the first moments of (M_0, P_0) and (M_{τ_R}, P_{τ_R}) so that the mean and variance of protein number will be fully characterized.

Description of the Process of the Number of Proteins Before beginning, we describe the number of proteins P_t at any time t. We will use this description in the following proofs. Similarly to mRNA case Equation (2.1), we group them into two categories.

— The P_0 proteins that were there at birth and which remain in the cell during all the cell cycle (as said in the main article the proteolysis is not considered in this model).

— The proteins that were created during the cell cycle. The rate of production depends on the current number of mRNAs. For that we consider $\left(\mathcal{N}^i_{\lambda_2}\right)_{i\in\mathbb{N}}$, a sequence of i.i.d. Poisson Point Processes of intensity λ_2; if the i-th mRNA exists at time t (that is to say if $i \leq M_t$), then the number of proteins produced by this mRNA between t and $t + \mathrm{d}t$ is $\mathcal{N}^i_{\lambda_2}(\mathrm{d}t)$. Hence, the total number of proteins produced between t and $t + \mathrm{d}t$ is then $\sum_{i=1}^{\infty} \mathbf{1}_{\{i\leq M_u\}} \mathcal{N}^i_{\lambda_2}(\mathrm{d}t)$.

To summarize, the number of proteins at a time t of the cell cycle is

$$P_t = P_0 + \sum_{i=1}^{\infty} \int_0^t \mathbf{1}_{\{i\leq M_u\}} \mathcal{N}^i_{\lambda_2}(\mathrm{d}u)\,. \tag{2.2}$$

The first term is the number of proteins at birth, and the second takes into account all proteins created between times 0 and t.

Protein Number Before Replication We begin with the case before replication, $t < \tau_R$. We use the notation $\langle\cdot\rangle_{M_0,P_0}$ as the conditional expectation given (M_0, P_0), i.e. $\langle\cdot\rangle_{M_0,P_0} = \langle\cdot|\,(M_0, P_0)\rangle$. We first characterize the first two moments of P_t conditionally on (M_0, P_0). As for the mRNAs, we determine at first the moment-generating function of P_t.

Proposition 6. *For any $t \in [0, \tau_R[$, the conditional moment generating function of P_t can be expressed as*

$$\langle \exp(\xi P_t)\rangle_{M_0,P_0} = \exp(\xi P_0) \cdot h_t\left(\lambda_2\left(e^{\xi} - 1\right)\right)$$

for any $\xi < 0$ and such as h_t is the moment generating function of $\int_0^t M_u\,\mathrm{d}u$. The expression of h_t is given by

$$h_t(\xi) := \exp\left[M_0 \log\left[\frac{\sigma_1 - \xi e^{-(\sigma_1-\xi)t}}{\sigma_1 - \xi}\right] + \lambda_1 \frac{\xi}{\sigma_1 - \xi}\left(t - \frac{1 - e^{-(\sigma_1-\xi)t}}{\sigma_1 - \xi}\right)\right].$$

Proof. With Equation (2.2), it is easy to show that

$$\langle \exp(\xi P_t) \rangle_{M_0,P_0} = \exp(\xi P_0) \cdot \left\langle \prod_{i=1}^{\infty} \left\langle \exp\left(\xi \int_0^t \mathbf{1}_{\{i \le M_u\}} \mathcal{N}_{\lambda_2}^i(\mathrm{d}u)\right) \mid (M_u)_{u \le t} \right\rangle_{M_0,P_0} \right\rangle_{M_0,P_0}.$$

We then consider the Laplace functional of the Poisson process $\mathcal{N}_{\lambda_2}^i$,

$$\left\langle \exp\left(\xi \int_0^t \mathbf{1}_{\{i \le M_u\}} \mathcal{N}_{\lambda_2}^i(\mathrm{d}u)\right) \middle| (M_u)_{u \le t}, P_0 \right\rangle = \exp\left[\lambda_2 \int_0^{\infty} \left(\exp\left(\xi \mathbf{1}_{\{i \le M_u\}} \mathbf{1}_{\{u \le t\}}\right) - 1\right) du\right]$$
$$= \exp\left[\lambda_2 \left(e^{\xi} - 1\right) \int_0^t \mathbf{1}_{\{i \le M_u\}} du\right].$$

By making the product for i from 1 to infinity, one gets

$$\prod_{i=1}^{\infty} \left\langle \exp\left(\xi \int_0^t \mathbf{1}_{\{i \le M_u\}} \mathcal{N}_{\lambda_2}^i(\mathrm{d}u)\right) \mid (M_u)_{u \le t} \right\rangle_{M_0,P_0} = \exp\left[\lambda_2 \left(e^{\xi} - 1\right) \int_0^t M_u du\right].$$

As a consequence, it indeed follows that

$$\langle \exp(\xi P_t) \rangle_{M_0,P_0} = \exp(\xi P_0) \cdot h_t\left(\lambda_2\left(e^{\xi} - 1\right)\right).$$

Using the expression (2.1) of M_t, integrated between time 0 and $t < \tau_R$ gives the result. For more details of the calculations, see Chapter 3 of [7]). □

As the moment generating function of P_t has been characterized, it is possible to deduce, by derivation, the first two moments of P_t knowing (M_0, P_0) for any time t before the gene replication.

Corollary 7. *At steady state, for $t \in [0, \tau_R[$, the first two conditional moments of P_t are given by*

$$\langle P_t \rangle_{M_0,P_0} = P_0 + \lambda_2 \left(\frac{\lambda_1}{\sigma_1} t + \left(M_0 - \frac{\lambda_1}{\sigma_1}\right) \frac{1 - e^{-\sigma_1 t}}{\sigma_1}\right),$$
$$\langle P_t^2 \rangle_{M_0,P_0} = \left(\langle P_t \rangle_{M_0,P_0}\right)^2 + M_0 \frac{\lambda_2}{\sigma_1} \left(1 - e^{-\sigma_1 t} + \frac{\lambda_2}{\sigma_1}\left[1 - e^{-\sigma_1 t}\left(e^{-\sigma_1 t} + 2t\sigma_1\right)\right]\right)$$
$$+ \frac{\lambda_1 \lambda_2}{\sigma_1^2}\left[t\sigma_1 - 1 + e^{-\sigma_1 t} + 2\frac{\lambda_2}{\sigma_1}\left(\sigma_1 t\left(1 + e^{-\sigma_1 t}\right) - 2\left(1 - e^{-\sigma_1 t}\right)\right)\right]$$

Proof. The first two moments of P_t can be obtained by derivation of the moment generating function of Proposition 6,

$$\langle P_t \rangle_{M_0,P_0} = \lim_{\xi \to 0} \frac{d}{d\xi}\left[\exp(\xi P_0) h_t\left(\lambda_2\left(e^{\xi} - 1\right)\right)\right] = P_0 + \lambda_2 h_t'(0)$$

and

$$\langle P_t^2 \rangle_{M_0,P_0} = \lim_{\xi \to 0} \frac{d^2}{d\xi^2}\left[\exp(\xi P_0) h_t\left(\lambda_2\left(e^{\xi} - 1\right)\right)\right]$$
$$= \left(\langle P_t \rangle_{M_0,P_0}\right)^2 + \lambda_2 h_t'(0) + (\lambda_2)^2 \left(h_t''(0) - h_t'(0)^2\right)$$

The calculations of $h_t'(0)^2$, $h_t''(0)^2$ allow to show the result (see Chapter 3 of [7] for the details of the calculation). □

The previous corollary gives expressions for $\langle P_t \rangle_{M_0,P_0}$ and $\langle P_t^2 \rangle_{M_0,P_0}$. In the next proposition, we integrate these expressions over all birth states (M_0, P_0) to find formulas for $\langle P_t \rangle$ and $\mathrm{Var}[P_t]$ for any time $t < \tau_R$ before replication. These expression depends on joint moments of M_0 and P_0.

Proposition 8. *At any time $t \in [0, \tau_R[$ before replication, the mean and the variance of P_t are given by*

$$\langle P_t \rangle = \langle P_0 \rangle + \lambda_2 \left(\frac{\lambda_1}{\sigma_1} t + \left(x_0 - \frac{\lambda_1}{\sigma_1} \right) \frac{1 - e^{-\sigma_1 t}}{\sigma_1} \right),$$

$$\begin{aligned}\mathrm{Var}\left[P_t\right] &= \mathrm{Var}\left[P_0\right] + 2\lambda_2 \frac{1 - e^{-\sigma_1 t}}{\sigma_1} \mathrm{Cov}\left[P_0, M_0\right] + \left(\lambda_2 \frac{1 - e^{-\sigma_1 t}}{\sigma_1} \right)^2 x_0 \\ &+ x_0 \frac{\lambda_2}{\sigma_1} \left(1 - e^{-\sigma_1 t} + \frac{\lambda_2}{\sigma_1} \left[1 - e^{-\sigma_1 t} \left(e^{-\sigma_1 t} + 2t\sigma_1 \right) \right] \right) \\ &+ \frac{\lambda_1 \lambda_2}{\sigma_1^2} \left[t\sigma_1 - 1 + e^{-\sigma_1 t} + 2\frac{\lambda_2}{\sigma_1} \left(\sigma_1 t \left(1 + e^{-\sigma_1 t} \right) - 2\left(1 - e^{-\sigma_1 t} \right) \right) \right]\end{aligned}$$

where x_0 is defined in Proposition 4.

Proof. By considering the mean of the random variable $\langle P_t | (M_0, P_0) \rangle$ in Corollary 7, the result for $\langle P_t \rangle$ is easy to get. For the variance, consider the expression of $\left\langle P_t^2 | (M_0, P_0) \right\rangle$

$$\begin{aligned}\left\langle P_t^2 \right\rangle = \left\langle \langle P_t \rangle_{M_0, P_0}^2 \right\rangle + \langle M_0 \rangle \frac{\lambda_2}{\sigma_1} \left(1 - e^{-\sigma_1 t} + \frac{\lambda_2}{\sigma_1} \left[1 - e^{-\sigma_1 t} \left(e^{-\sigma_1 t} + 2t\sigma_1 \right) \right] \right) \\ + \frac{\lambda_1 \lambda_2}{\sigma_1^2} \left[t\sigma_1 - 1 + e^{-\sigma_1 t} + 2\frac{\lambda_2}{\sigma_1} \left(\sigma_1 t \left(1 + e^{-\sigma_1 t} \right) - 2\left(1 - e^{-\sigma_1 t} \right) \right) \right]\end{aligned}$$

and

$$\begin{aligned}\mathrm{Var}\left[P_t\right] = \left\langle \langle P_t \rangle_{M_0, P_0}^2 \right\rangle - \langle P_t \rangle^2 + \langle M_0 \rangle \frac{\lambda_2}{\sigma_1} \left(1 - e^{-\sigma_1 t} + \frac{\lambda_2}{\sigma_1} \left[1 - e^{-\sigma_1 t} \left(e^{-\sigma_1 t} + 2t\sigma_1 \right) \right] \right) \\ + \frac{\lambda_1 \lambda_2}{\sigma_1^2} \left[t\sigma_1 - 1 + e^{-\sigma_1 t} + 2\frac{\lambda_2}{\sigma_1} \left(\sigma_1 t \left(1 + e^{-\sigma_1 t} \right) - 2\left(1 - e^{-\sigma_1 t} \right) \right) \right].\end{aligned}$$

Now, for the expression of $\left\langle \langle P_t \rangle_{M_0, P_0}^2 \right\rangle - \langle P_t \rangle^2$,

$$\begin{aligned}\left\langle \langle P_t \rangle_{M_0, P_0}^2 \right\rangle - \langle P_t \rangle^2 &= \left\langle P_0^2 \right\rangle + \left\langle \left(\lambda_2 \left(\frac{\lambda_1}{\sigma_1} t + \left(M_0 - \frac{\lambda_1}{\sigma_1} \right) \frac{1 - e^{-\sigma_1 t}}{\sigma_1} \right) \right)^2 \right\rangle \\ &+ 2 \left\langle P_0 \times \lambda_2 \left(\frac{\lambda_1}{\sigma_1} t + \left(M_0 - \frac{\lambda_1}{\sigma_1} \right) \frac{1 - e^{-\sigma_1 t}}{\sigma_1} \right) \right\rangle \\ &- \langle P_0 \rangle^2 + \left\langle \lambda_2 \left(\frac{\lambda_1}{\sigma_1} t + \left(M_0 - \frac{\lambda_1}{\sigma_1} \right) \frac{1 - e^{-\sigma_1 t}}{\sigma_1} \right) \right\rangle^2 \\ &- 2 \langle P_0 \rangle \left\langle \lambda_2 \left(\frac{\lambda_1}{\sigma_1} t + \left(M_0 - \frac{\lambda_1}{\sigma_1} \right) \frac{1 - e^{-\sigma_1 t}}{\sigma_1} \right) \right\rangle \\ &= \mathrm{Var}\left[P_0\right] + \mathrm{Var}\left[\lambda_2 \left(\frac{\lambda_1}{\sigma_1} t + \left(M_0 - \frac{\lambda_1}{\sigma_1} \right) \frac{1 - e^{-\sigma_1 t}}{\sigma_1} \right) \right] \\ &\quad + 2\mathrm{Cov}\left[P_0, \lambda_2 \left(\frac{\lambda_1}{\sigma_1} t + \left(M_0 - \frac{\lambda_1}{\sigma_1} \right) \frac{1 - e^{-\sigma_1 t}}{\sigma_1} \right) \right].\end{aligned}$$

Finally, one just has to remark that due to Proposition 4 $\langle M_0 \rangle = \mathrm{Var}\left[M_0\right] = x_0$. □

Protein Number After Replication For a time t such as $\tau_R \le t < \tau_D$. We adopt a similar approach as for the previous case, the state just after replication (M_{τ_R}, P_{τ_R}) is known, and we want to determine the first two moments of P_t for any time t after the replication.

Proposition 9. *At steady state, for a time* $t \in [\tau_R, \tau_D[$*, conditionally on the state of the cell at replication* (M_{τ_R}, P_{τ_R})*, the first two moments of* P_t *are given by*

$$\langle P_t \rangle_{M_{\tau_R}, P_{\tau_R}} = P_{\tau_R} + \lambda_2 \left(2\frac{\lambda_1}{\sigma_1} (t - \tau_R) + \left(M_{\tau_R} - 2\frac{\lambda_1}{\sigma_1} \right) \frac{1 - e^{-\sigma_1 (t - \tau_R)}}{\sigma_1} \right),$$

$$\begin{aligned}
\langle P_t^2 \rangle_{M_{\tau_R}, P_{\tau_R}} &= \left(\langle P_t \rangle_{M_{\tau_R}, P_{\tau_R}} \right)^2 \\
&+ M_{\tau_R} \frac{\lambda_2}{\sigma_1} \left(1 - e^{-\sigma_1 (t - \tau_R)} + \frac{\lambda_2}{\sigma_1} \left[1 - e^{-\sigma_1 (t - \tau_R)} \left(e^{-\sigma_1 (t - \tau_R)} + 2 (t - \tau_R) \sigma_1 \right) \right] \right) \\
&+ 2\frac{\lambda_1 \lambda_2}{\sigma_1^2} \left[(t - \tau_R) \sigma_1 - 1 + e^{-\sigma_1 (t - \tau_R)} + \right. \\
&\qquad \left. 2\frac{\lambda_2}{\sigma_1} \left(\sigma_1 (t - \tau_R) \cdot \left(1 + e^{-\sigma_1 (t - \tau_R)} \right) - 2 \left(1 - e^{-\sigma_1 (t - \tau_R)} \right) \right) \right].
\end{aligned}$$

Proof. After the replication, the rate of mRNA production is doubled, but otherwise, the dynamic is identical as it was before the replication. One can hence easily adapt the proofs of Proposition 6 and Corollary 7, by replacing the initial state by the state at replication (M_{τ_R}, P_{τ_R}), by considering that the mRNA production rate is $2\lambda_1$, and that the time spent since the initial state is $t - \tau_R$. □

We can then integrate the previous expressions on all possible states at replication (M_{τ_R}, P_{τ_R}). It follows that

Proposition 10. *At any time* $t \in [\tau_R, \tau_D[$ *after replication, depending on joint moments of* P_{τ_R} *and* M_{τ_R}*, the mean and the variance of* P_t *are given by*

$$\langle P_t \rangle = \langle P_{\tau_R} \rangle + \lambda_2 \left(2\frac{\lambda_1}{\sigma_1} (t - \tau_R) + \left(x_{\tau_R} - 2\frac{\lambda_1}{\sigma_1} \right) \frac{1 - e^{-\sigma_1 (t - \tau_R)}}{\sigma_1} \right),$$

$$\begin{aligned}
\mathrm{Var}[P_t] &= \mathrm{Var}[P_{\tau_R}] + 2\lambda_2 \frac{1 - e^{-\sigma_1 (t - \tau_R)}}{\sigma_1} \mathrm{Cov}[P_{\tau_R}, M_{\tau_R}] + \left(\lambda_2 \frac{1 - e^{-\sigma_1 (t - \tau_R)}}{\sigma_1} \right)^2 x_{\tau_R} \\
&+ x_{\tau_R} \frac{\lambda_2}{\sigma_1} \left(1 - e^{-\sigma_1 (t - \tau_R)} + \frac{\lambda_2}{\sigma_1} \left[1 - e^{-\sigma_1 (t - \tau_R)} \left(e^{-\sigma_1 (t - \tau_R)} + 2 (t - \tau_R) \sigma_1 \right) \right] \right) \\
&+ 2\frac{\lambda_1 \lambda_2}{\sigma_1^2} \left[(t - \tau_R) \sigma_1 - 1 + e^{-\sigma_1 (t - \tau_R)} \right. \\
&\qquad \left. + 2\frac{\lambda_2}{\sigma_1} \left(\sigma_1 (t - \tau_R) \left(1 + e^{-\sigma_1 (t - \tau_R)} \right) - 2 \left(1 - e^{-\sigma_1 (t - \tau_R)} \right) \right) \right],
\end{aligned}$$

with x_{τ_R} *as defined in Theorem 5.*

Proof. It is similar to the proof of Proposition 8. □

Protein Number in the Whole Cell Cycle In order to have an analytic expression for the mean $\langle P_t \rangle$ and variance $\mathrm{Var}[P_t]$ for any time t of the cell cycle, we need to have expressions for the means $\langle P_0 \rangle$ and $\langle P_{\tau_R} \rangle$, the variances $\mathrm{Var}[P_0]$ and $\mathrm{Var}[P_{\tau_R}]$ as well as the covariances $\mathrm{Cov}[P_0, M_0]$ and $\mathrm{Cov}[P_{\tau_R}, M_{\tau_R}]$. The general idea is to use the steady state properties that give a relation between the distributions at birth and at division. Indeed, it gives:

$$P_{\tau_D} \overset{\mathcal{D}}{=} P_0 \quad \text{and} \quad (M_{\tau_D}, P_{\tau_D}) \overset{\mathcal{D}}{=} (M_0, P_0).$$

Indeed, between times τ_D- and τ_D, the proteins undergo a random partitioning, and since the system is at steady state, the distribution of the number of proteins after division P_{τ_D} is the same as the distribution of proteins at birth P_0. As a consequence:

$$\sum_{i=1}^{P_{\tau_D-}} B_{i,1/2} \overset{\mathcal{D}}{=} P_0$$

with $\left(B_{i,1/2}\right)$ being independent Bernoulli random variables of parameter 1/2 and being all independent of P_{τ_D-}.

Lemma 11. *The mean and the variance of P_0 depend on the mean and the variance of P_{τ_D-} in the following way*

$$\langle P_{\tau_D-}\rangle = 2\langle P_0\rangle \qquad \mathrm{Var}\left[P_{\tau_D-}\right] = 4\mathrm{Var}\left[P_0\right] - 2\langle P_0\rangle .$$

Proof. With the moment-generating function of P_0, one gets

$$\langle \exp\left[\xi P_0\right]\rangle = \left\langle \prod_{1=1}^{P_{\tau_D-}} \left\langle \exp\left[B_{i,1/2}\right]\right\rangle \right\rangle = \left\langle \left(\frac{1+e^{\xi}}{2}\right)^{P_{\tau_D-}} \right\rangle = \left\langle \exp\left[\log\left(\frac{1+e^{\xi}}{2}\right) P_{\tau_D-}\right]\right\rangle$$

As a consequence, by denoting $\eta(\xi) := \langle \exp\left[\xi P_{\tau_D-}\right]\rangle$ the moment generating function of P_{τ_D-}, it follows:

$$\frac{\mathrm{d}}{\mathrm{d}\xi}\langle \exp\left[\xi P_0\right]\rangle = \frac{e^{\xi}}{1+e^{\xi}} \cdot \eta'\left(\log\left(\frac{1+e^{\xi}}{2}\right)\right)$$
$$\frac{\mathrm{d}^2}{\mathrm{d}\xi^2}\langle \exp\left[\xi P_0\right]\rangle = \frac{e^{\xi}}{\left(1+e^{\xi}\right)^2} \cdot \eta'\left(\log\left(\frac{1+e^{\xi}}{2}\right)\right) + \left(\frac{e^{\xi}}{1+e^{\xi}}\right)^2 \cdot \eta''\left(\log\left(\frac{1+e^{\xi}}{2}\right)\right).$$

As ξ goes to 0, one getss

$$\langle P_0\rangle = \frac{\langle P_{\tau_D-}\rangle}{2} \text{ and } \left\langle P_0^2\right\rangle = \frac{1}{4}\cdot\langle P_{\tau_D-}\rangle + \frac{1}{4}\cdot\left\langle P_{\tau_D-}^2\right\rangle .$$

The lemma is proved. □

We then use this Lemma to calculate the means $\langle P_0\rangle$ and $\langle P_{\tau_R}\rangle$ and the variances $\mathrm{Var}\left[P_0\right]$ and $\mathrm{Var}\left[P_{\tau_R}\right]$.

Proposition 12. *For $\eta = 1, 2$, denote,*

$$f_\eta(t) := \eta\frac{\lambda_1}{\sigma_1}(t-\tau) + \left(x_\tau - \eta\frac{\lambda_1}{\sigma_1}\right)\frac{1-e^{-\sigma_1(t-\tau)}}{\sigma_1}$$

with $\tau = 0$ in the case of $\eta = 1$ (before replication) and $\tau = \tau_R$ for the case $\eta = 2$ (after replication). In that case, we have that:

$$\langle P_0\rangle = \lambda_2\left(f_1\left(\tau_R\right) + f_2\left(\tau_D\right)\right) \text{ and } \langle P_{\tau_R}\rangle = \lambda_2\left(2f_1\left(\tau_R\right) + f_2\left(\tau_D\right)\right).$$

Proof. With Propositions 8 and 10, one gets

$$\langle P_{\tau_D}\rangle = \langle P_{\tau_R}\rangle + \lambda_2\left(f_2\left(\tau_D\right)\right) = \langle P_0\rangle + \lambda_2\left(f_1\left(\tau_R\right) + f_2\left(\tau_D\right)\right).$$

We conclude with the Lemma 11. □

Proposition 13. *For* $\eta = 1, 2$*, define*

$$\begin{aligned} g_\eta(t) := & \left(\lambda_2 \frac{1-e^{-\sigma_1 (t-\tau)}}{\sigma_1}\right)^2 x_\tau \\ & + x_\tau \frac{\lambda_2}{\sigma_1}\left(1 - e^{-\sigma_1(t-\tau)} + \frac{\lambda_2}{\sigma_1}\left[1 - e^{-\sigma_1(t-\tau)}\left(e^{-\sigma_1(t-\tau)} + 2\left(t-\tau\right)\sigma_1\right)\right]\right) \\ + \eta \frac{\lambda_1 \lambda_2}{\sigma_1^2} & \left[\left(t-\tau\right)\sigma_1 - 1 + e^{-\sigma_1(t-\tau)} + 2\frac{\lambda_2}{\sigma_1}\left(\sigma_1\left(t-\tau\right)\left(1+e^{-\sigma_1(t-\tau)}\right) - 2\left(1 - e^{-\sigma_1(t-\tau)}\right)\right)\right]. \end{aligned}$$

with $\tau = 0$ *in the case of* $\eta = 1$ *(before replication) and* $\tau = \tau_R$ *for the case* $\eta = 2$ *(after replication). In that case, we have that:*

$$\begin{aligned} \mathrm{Var}\left[P_0\right] = & \frac{1}{3}\left\{ 2\left\langle P_0 \right\rangle + \right. \\ & 2\frac{\lambda_2}{\sigma_1}\left[\left(1 - e^{-\sigma_1 \tau_R}\right)\mathrm{Cov}\left[P_0, M_0\right] + \left(1 - e^{-\sigma_1(\tau_D - \tau_R)}\right)\mathrm{Cov}\left[P_{\tau_R}, M_{\tau_R}\right]\right] \\ & \left. + g_1\left(\tau_R\right) + g_2\left(\tau_D\right)\right\}. \end{aligned}$$

Proof. By considering the expressions of Proposition 10 for $t = \tau_D-$,

$$\mathrm{Var}\left[P_{\tau_D -}\right] = \mathrm{Var}\left[P_{\tau_R}\right] + 2\lambda_2 \frac{1 - e^{-\sigma_1(\tau_D - \tau_R)}}{\sigma_1}\mathrm{Cov}\left[P_{\tau_R}, M_{\tau_R}\right] + g_2\left(\tau_D\right).$$

Similarly, the expression of Proposition 8 for $t = \tau_R-$ gives the expression of $\mathrm{Var}\left[P_{\tau_R}\right]$ by continuity. We have

$$\begin{aligned} \mathrm{Var}\left[P_{\tau_D -}\right] = & \mathrm{Var}\left[P_0\right] + 2\frac{\lambda_2}{\sigma_1}\left[\left(1 - e^{-\sigma_1 \tau_R}\right)\mathrm{Cov}\left[P_0, M_0\right] + \left(1 - e^{-\sigma_1(\tau_D - \tau_R)}\right)\mathrm{Cov}\left[P_{\tau_R}, M_{\tau_R}\right]\right] \\ & + g_1\left(\tau_R\right) + g_2\left(\tau_D\right). \end{aligned}$$

Lemma 11 describes the effect of the binomial sampling between τ_D- and τ_D on the mean and the variance of P. Since, we are at steady state of cell cycles, one has

$$\begin{aligned} 3\mathrm{Var}\left[P_0\right] = & 2\left\langle P_0 \right\rangle + 2\frac{\lambda_2}{\sigma_1}\left[\left(1 - e^{-\sigma_1 \tau_R}\right)\mathrm{Cov}\left[P_0, M_0\right] + \left(1 - e^{-\sigma_1(\tau_D - \tau_R)}\right)\mathrm{Cov}\left[P_{\tau_R}, M_{\tau_R}\right]\right] \\ & + g_1\left(\tau_R\right) + g_2\left(\tau_D\right). \end{aligned}$$

□

The expression of $\mathrm{Var}\left[P_{\tau_R}\right]$ can then be deduced from Proposition 8.

Proposition 14. *For* $\eta = 1, 2$*, define*

$$\begin{aligned} k_\eta\left(t\right) := & \frac{\eta \lambda_1 \lambda_2}{\sigma_1^2}\left\langle M_\tau \right\rangle e^{-(t-\tau)\sigma_1}\left(\left(t-\tau\right)\sigma_1 - \left(1 - e^{-\sigma_1(t-\tau)}\right)\right) \\ & + \frac{\eta \lambda_1}{\sigma_1}\left\langle P_\tau \right\rangle\left(1 - e^{-(t-\tau)\sigma_1}\right) + \frac{\eta \lambda_1 \lambda_2}{\sigma_1^2}\left\langle M_\tau \right\rangle\left(1 - e^{-(t-\tau)\sigma_1}\right)^2 \\ & + \frac{\lambda_2}{\sigma_1} e^{-(t-\tau)\sigma_1}\left(\left(\left\langle M_\tau^2 \right\rangle - \left\langle M_\tau \right\rangle\right)\left(1 - e^{-\sigma_1(t-\tau)}\right) + \sigma_1\left(t-\tau\right)\left\langle M_\tau \right\rangle\right) \\ & + \frac{\eta \lambda_1 \lambda_2}{\sigma_1^2}\left[\frac{\eta \lambda_1}{\sigma_1}\left(1 - e^{-(t-\tau)\sigma_1}\right)\left(\left(t-\tau\right)\sigma_1 - \left(1 - e^{-\sigma_1(t-\tau)}\right)\right) + \right. \\ & \left. \left(1 - e^{-\sigma_1(t-\tau)}\left(\left(t-\tau\right)\sigma_1 + 1\right)\right)\right]. \end{aligned}$$

with $\tau = 0$ in the case of $\eta = 1$ (before replication) and $\tau = \tau_R$ for the case $\eta = 2$ (after replication). In that case, the covariances can be expressed as

$$\mathrm{Cov}\left[M_0, P_0\right] = \frac{1}{\left(4 - e^{-\tau_D \sigma_1}\right)} \left\{ k_1\left(\tau_R\right) e^{-(\tau_D - \tau_R)\sigma_1} + k_2\left(\tau_D\right) \right\} - \left\langle M_0 \right\rangle \left\langle P_0 \right\rangle$$

and

$$\mathrm{Cov}\left[M_{\tau_R}, P_{\tau_R}\right] = \left(\mathrm{Cov}\left[M_0, P_0\right] + \left\langle M_0 \right\rangle \left\langle P_0 \right\rangle\right) e^{-\tau_R \sigma_1} + k_1\left(\tau_R\right) - \left\langle M_{\tau_R} \right\rangle \left\langle P_{\tau_R} \right\rangle .$$

Proof. The proof follows the same arguments as in the proofs of the previous propositions. Details of the calculations can be found in Chapter 3 of [7]. □

2.2 Parameter Estimation

As in the previous intermediate model, we set the doubling time τ_D to 150 min and the volume at birth $V_0 = 1.3\,\mu\mathrm{m}^3$. For each gene, we have to determine four different parameters λ_1, σ_1, λ_2 and τ_R. We have considered the genes of [1] for which the empirical mean of messengers μ_m and proteins μ_p concentrations, as well as the mRNA half-life time τ_m have been measured. We still deduce the mRNA degradation rate σ_1 with the mRNA half-life time τ_m (such that $\sigma_1 = \log 2/\tau_m$).

2.2.1 A Model for the Instants of Gene Replication

In this model, the time at which each gene is replicated is estimated as follows: we first determine the time of DNA replication initiation (the time τ_I in the cell cycle); as we consider that the DNA-polymerase replicates DNA at constant speed, we can deduce the time of replication of each gene only by knowing its position in the DNA.

The article [8] investigates the replication initiation. It is shown that the initiation occurs at a fixed volume per replication origin, and thus independently from the time since the previous division. Furthermore, this volume seems to be constant for different conditions. For slow growing bacteria (with only one DNA replication per cell cycle), such as those in [1], the volume at which DNA replication initiation occurs is $V_I = 1.8\,\mu\mathrm{m}^3$. As in our model, the volume is considered as growing exponentially, we define the time of replication initiation τ_I as

$$\tau_I = \frac{\tau_D}{\log 2} \log \frac{V_I}{V_0}.$$

The initiation of DNA replication occurs at τ_I, the remaining delay to gene replication of each gene is considered as deterministic (we consider the speed of DNA replication as constant). The whole chromosome is replicated in around 40 min [9], therefore the distance of the gene from the origin of replication is sufficient to determine the time it takes for the DNA-polymerase to replicate it. The position of each gene was determined with Ecogene database [10].

2.2.2 Estimation of λ_1 and λ_2 in an Homogeneous Population

We still have to determine the rates λ_1 and λ_2. One can interpret the empirical average mRNA and protein concentration of the experiment (respectively μ_m and μ_p) as the global average of mRNA and protein concentrations of the model (respectively $\overline{\langle M/V \rangle}$ and $\overline{\langle P/V \rangle}$).

Contrary to the previous intermediate model, the mean concentrations $\langle M_t/V(t) \rangle$ and $\langle P_t/V(t) \rangle$ change during the cell cycle. As depicted in Equation (6) of the main article, in order to consider the concentrations $\overline{\langle M/V \rangle}$ and $\overline{\langle P/V \rangle}$ averaged over the cell

population, one have to explicit the age distribution u of the population. We consider at first that the distribution is homogeneous between age 0 and τ_D (see Section 2.2.3 for a more realistic distribution). Then, the global averages are known through the integration over the cell cycle of the mean formulas of Theorem 5 and the Propositions 8 and 10 we can write the global average of mRNA and protein concentrations as

$$\overline{\langle M/V\rangle} = \frac{\lambda_1}{\sigma_1}\frac{1}{\tau_D}\int_0^{\tau_D}\frac{1}{V_0 2^{t/\tau_D}}\left(1-\frac{e^{-(t+\tau_D-\tau_R)\sigma_1}}{2-e^{-\tau_D\sigma_1}}+\mathbf{1}_{\{t\geq\tau_R\}}\left(1-e^{-(t-\tau_R)\sigma_1}\right)\right)\mathrm{d}t,$$
$$\overline{\langle P/V\rangle} = \lambda_2\frac{1}{\tau_D}\int_0^{\tau_D}\frac{1}{V_0 2^{t/\tau_D}}\left(f_1(\tau_R)+f_2(\tau_D)+f_1(\tau_R\wedge t)+\mathbf{1}_{\{t\geq\tau_R\}}f_2(t)\right)\mathrm{d}t.$$

As a consequence, parameters λ_1 and λ_2 can be expressed as follows:

$$\lambda_1 = \sigma_1\tau_D\mu_m\left(\int_0^{\tau_D}\frac{1}{V_0 2^{t/\tau_D}}\left(1-\frac{e^{-(t+\tau_D-\tau_R)\sigma_1}}{2-e^{-\tau_D\sigma_1}}+\mathbf{1}_{\{t\geq\tau_R\}}\left(1-e^{-(t-\tau_R)\sigma_1}\right)\right)\mathrm{d}t\right)^{-1},$$
$$\lambda_2 = \tau_D\mu_p\left(\int_0^{\tau_D}\frac{1}{V_0 2^{t/\tau_D}}\left(f_1(\tau_R)+f_2(\tau_D)+f_1(\tau_R\wedge t)+\mathbf{1}_{\{t\geq\tau_R\}}f_2(t)\right)\mathrm{d}t\right)^{-1}.$$

For each gene, all parameters can be hence determined.

2.2.3 Impact of the Distribution of the Population of Cells

As previously noticed, the definitions of $\overline{\langle M/V\rangle}$ and $\overline{\langle P/V\rangle}$ depends on the population age distribution. In real experimental populations of cells (like in [1]) the number of cells in the population is exponentially growing: any dividing cell gives birth to two daughter cells. The distribution of ages is therefore not uniform.

Using a classic age distribution u in the definitions $\overline{\langle M/V\rangle}$ and $\overline{\langle P/V\rangle}$ (Equation (6) of the main article) for exponentially growing populations (see [11–13] for instance), we have performed a parameter estimation that takes into account this effect. For any gene, protein variance is estimated in both cases: either with an uniform population or an exponentially growing population. The variances in both cases are almost identical (the histogram ratio of both variances is centered around 1 with a standard deviation of $8\cdot 10^{-3}$).

The distribution considered does not have a significant impact on the variance of the model. This is due to the fact that the mean concentration $\mathrm{Var}\left[P_t/V(t)\right]$ of any protein remains approximately constant during the cell cycle, there is therefore no significant difference of protein concentration dosage at the beginning or at the end of the cell cycle. We observe the same effect in the case of the complete model of next section (with the sharing of RNA-polymerases and ribosomes).

3 Impact of the Sharing of RNA-Polymerases and Ribosomes

3.1 A Detailed Description of the Model

The unit of production of one particular protein is presented in S7 Fig. We recall that, for any time t, the copy number of the i-th gene is $G_i(t)$, the number of mRNA is $M_i(t)$ and the number of proteins is $P_i(t)$, the number of RNA-polymerases sequestered on the i-th gene is $E_{Y,i}(t)$ and the number of ribosomes sequestered on an mRNA of type i is $E_{R,i}(t)$. The number of non-sequestered RNA-polymerases and ribosomes are respectively denoted as $F_Y(t)$ and $F_R(t)$.

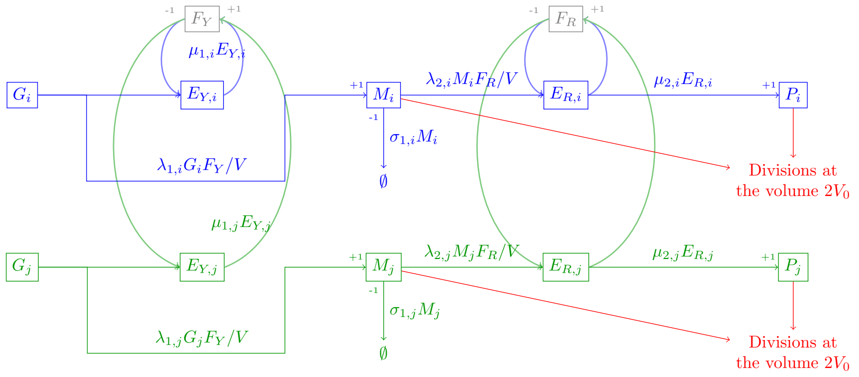

S7 Fig. Production unit of the i-th and j-th protein with the common pools of free RNA-polymerases and ribosomes.

Transcription In the current model, the process of mRNA production is considered as taking part in two steps: first, the binding of the RNA-polymerase and initiation; and second, the elongation and termination of the mRNA. For the first step, *inside a unit volume*, the rate at which an RNA-polymerase binds on the promoter of the i-th gene is given by the law of mass action

$$\lambda_{1,i}\frac{G_i(t)}{V(t)}\frac{F_Y(t)}{V(t)}.$$

with $\lambda_{1,i}$ accounts for the specificity of the promoter (its affinity for the RNA-polymerase, the chromosome conformation, etc.). As we are interested in the rate of reactions inside the whole cell of volume $V(t)$, the rate of reaction is then

$$\lambda_{1,i}G_i(t)\frac{F_Y(t)}{V(t)}.$$

The elongation time is given by an exponential random variable of rate $\mu_{1,i}$. Once the elongation terminates, the RNA-polymerase is released in the cytoplasm (increasing the number of free RNA-polymerases F_Y by one unit). A messenger is considered created as soon as its elongation begins: the reason for it is that in bacteria (unlike eukaryotes), since transcriptions and translations happen in the same medium, a translation can begin on an mRNA on which the transcription is not finished. As for the previous models, each messenger of type i has a lifetime given by an exponential random variable of rate $\sigma_{1,i}$.

Translation Similarly to the transcription, the rate at which a ribosome encounters an mRNA of type i and initiate translation is $\lambda_{2,i}M_i(t)F_R(t)/V(t)$ where $\lambda_{2,i}$ will account for mRNA specific aspects (RBS affinity for ribosomes, etc.). The total number of ribosomes sequestered on messengers of type i is $E_{Y,i}(t)$ and each elongation time follows an exponential distribution of rate $\mu_{2,i}$. Here we consider that the protein is created after the termination (since the protein is usually fully functional once its translation is completed); the number of proteins $P_i(t)$ is then increased by one unit. As previously we do not consider protein proteolysis since it usually occurs at much longer timescale than cell cycle.

DNA Replication and Division of the Cell At a time t, each gene $i \in \{1, ..., K\}$ is characterized by the gene copy number $G_i(t)$. As previously only one DNA replication per cell cycle is considered: as a consequence then, for each $i \in \{1, ..., K\}$, $G_i(t)$ is constant and equal to 1 (before replication) or to 2 (after

replication). There is two modeling choice for when the DNA replication is initiated: it can occur at a fixed time after the last division or when the cell reaches a certain volume V_I. The first simulations are made by considering the volume-dependent initiation event, but as we will see in Section 3.6.5, simulations with the other modeling choice show no noticeable difference. The volume V_I is fixed to $1.8\,\mu\text{m}^3$ (see [8] and Section 2.2.1 about this choice). We consider the speed of DNA replication as constant; as a consequence, once known the replication time τ_I, the delay until the replication of i-th gene is fixed, and is given by the gene position.

For the division, we considered at first that, like in the previous models, the division occurs when the cell reaches exactly the volume $2V_0$ (with $V_0 = 1.3\,\mu\text{m}^3$ as it was the case for the intermediate models considered earlier). We will consider in Section 3.6.4 the case where the division timing is not as precise. As before, the effect of septation is a random sampling of messengers and proteins: each of them has an equal chance to be in the next considered cell or not. Moreover, at division, all genes have only one copy.

Volume Increase As said in the main article, the volume $V(t)$ is no longer deterministic as it was the case in the previous intermediate models and it is considered as proportional to the current total mass of proteins in the cell. We denote by β_P represents ratio mass-volume and by w_i the mass of a type i protein. In that case, we have by definition

$$V(t) = \sum_{i=1}^{K} w_i P_i(t)/\beta_P. \tag{3.1}$$

Thus each protein of type i created increases the total volume of the cell with respect to the factor w_i/β_P. The mass w_i of a protein is determined according to its gene length.

Production of RNA-polymerases and ribosomes The total number of RNA-polymerases and ribosomes (whether allocated or not) are respectively denoted by $N_Y(t)$ and $N_R(t)$. In a first step, we consider that the both these quantities are in constant concentration, that is to say

$$N_Y(t) = \lfloor \beta_Y V(t) \rfloor \quad \text{and} \quad N_R(t) = \lfloor \beta_R V(t) \rfloor,$$

with β_Y and β_R constant parameters and where $\lfloor . \rfloor$ is the notation for the floor function. As the cell grows, new RNA-polymerases and ribosomes are added to the system in the corresponding proportion. When division occurs, ribosomes and RNA-polymerases will be set accordingly to the new volume. In Section 3.6 we will consider the more complex case where both RNA-polymerases and ribosomes are directly produced through a gene expression process.

3.2 Theoretical Analysis

This complete model is more complex than the previous ones. It is due in part to the feedback loop that proteins have on their own production: the more proteins, the more the volume increases, thereby increasing the total amount of ribosomes and hence the translation rates. This complicates the complete analytical description of mRNA and protein mean productions. In this section, we propose a description that mimics the average behavior of our stochastic model: the goal is to be able to fit parameters to real measures and use them for stochastic simulations.

3.2.1 Presentation of the Deterministic Production Model

The description chosen to reflect the average behavior of the stochastic model previously described is a system of ordinary differential equations (ODEs) that describes the kinetics of each compound concentration of the system.

We consider K genes, each of them has a corresponding type of mRNA and protein. For a gene of type i, the concentration of gene copies is given by $g_i(t)$; mRNAs and protein concentrations are denoted by $m_i(t)$ and $p_i(t)$. Similarly, $f_Y(t)$ and $f_R(t)$ respectively represent the concentrations of free RNA-polymerases and free ribosomes; while $e_{Y,i}(t)$ and $e_{R,i}(t)$ denote the concentrations of RNA-polymerases and ribosomes currently sequestered to produce type i proteins. All these quantities correspond to concentrations and not numbers of entities (their stochastic counterparts would be the concentrations $G_i(t)/V(t)$, $M_i(t)/V(t)$, $P_i(t)/V(t)$, etc.).

The reactions between different compounds are given by the *law of mass action*, that is to say that the rate of chemical reaction is proportional to the reactants abundance. We will study the evolution of m_i, the concentration of mRNAs of type i . The creation of a type i mRNA is the result of a reaction between a free RNA-polymerase (whose concentration is $f_Y(t)$) and the gene i (whose concentration is $g_i(t)$); $\lambda_{1,i}$ is interpreted as the affinity constant of the reaction. The type i mRNA degradation is the result of a reaction that occurs at rate $\sigma_{1,i}$.

As in the usual description of the cell (see [14] for instance), one also must consider the dilution: without any molecule creation, the concentration of the compound still decreases as the volume grows due to dilution. If we consider that cell volume is growing exponentially, doubling of volume in a time τ_D, then the rate of dilution is $\log 2/\tau_D$. The exponential growth corresponds to the volume dynamics of real bacteria [15], and we will see in Section 3.4 that it is a good approximation of the growth of cells in stochastic simulations.

All these aspects considered altogether, the kinetics of the concentration of mRNAs of type i is given by the ODE:

$$\frac{\mathrm{d}m_i}{\mathrm{d}t}(t) = \lambda_{1,i} g_i(t) \cdot f_Y(t) - \sigma_{1,i} m_i(t) - \frac{\log 2}{\tau_D} \cdot m_i(t). \tag{3.2}$$

The first term represents the mRNA creation; the second, the mRNA degradation; and the last, the dilution.

For the other reactions, for $i \in \{1, ..., K\}$, one has

$$\frac{\mathrm{d}p_i}{\mathrm{d}t}(t) = \mu_{2,i} e_{R,i}(t) - \frac{\log 2}{\tau_D} \cdot p_i(t), \tag{3.3}$$

$$\frac{\mathrm{d}e_{Y,i}}{\mathrm{d}t}(t) = \lambda_{1,i} g_i(t) \cdot f_Y(t) - \mu_{1,i} e_{Y,i}(t) - \frac{\log 2}{\tau_D} \cdot e_{Y,i}(t), \tag{3.4}$$

$$\frac{\mathrm{d}e_{R,i}}{\mathrm{d}t}(t) = \lambda_{2,i} m_i(t) \cdot f_R(t) - \mu_{2,i} e_{R,i}(t) - \frac{\log 2}{\tau_D} \cdot e_{R,i}(t). \tag{3.5}$$

As for the stochastic model of the previous section, assume that the concentration of RNA-polymerases (allocated or not) is constant and equal to β_Y, i.e.

$$\beta_Y = f_Y(t) + \sum_{i=1}^{K} e_{Y,i}(t), \tag{3.6}$$

since $\sum_i e_{Y,i}$ and f_Y represent the concentrations of respectively the allocated and non-allocated RNA-polymerases. It is similar to the ribosomes as we have:

$$\beta_R = f_R(t) + \sum_{i=1}^{K} e_{R,i}(t). \tag{3.7}$$

The classic strategy in literature to study such system (an analogous model is presented in [16]) is to consider the system in steady state growth: the gene concentration g_i is considered as constantly equal to its average value during the cell cycle, and then one can calculate the concentrations of m_i, p_i, $e_{Y,i}$ and $e_{R,i}$ at steady state by writing the Equations (3.2) to (3.7) with the derivative term as null. Using such method to determine parameters are unfortunatly not precise enough: there is a clear shift between the stochastic protein concentration and the one that shoule be obtained.

We have described the cell during one cycle with a non-constant gene concentration. The instant of replication of gene i replication within the cycle is denoted by $\tau_{R,i}$. in particular, at time t, the i-th gene copy number is known: $g_i(t) = \left(1 + \mathbf{1}_{\{t \geq \tau_{R,i}\}}\right)/(V_0 2^{t/\tau_D})$ (the factor $V_0 2^{t/\tau_D}$ represents the volume). By analogy with the steady state condition presented in Section 1.1.1, it is likely that a large number of cell cycles have already occurred, so that the concentration of any entities is the same at the beginning and at the end of the cell cycle. For each unit of production, the concentrations m_i, p_i, $e_{Y,i}$ and $e_{R,i}$ are such as

$$\forall i \in \{1, ..., K\} \quad \begin{cases} p_i(0) = p_i(\tau_D), & m_i(0) = m_i(\tau_D), \\ e_{Y,i}(0) = e_{Y,i}(\tau_D), & e_{R,i}(0) = e_{R,i}(\tau_D). \end{cases} \tag{3.8}$$

With these considerations, we have a system of ODEs to describe the average behavior of the main stochastic model during the cell cycle. In the next section, under some simplifications, we propose to give expressions for $m_i(t)$, $p_i(t)$, $e_{Y,i}(t)$, $e_{R,i}(t)$, $f_Y(t)$ and $f_R(t)$ as a function of all parameters ($\lambda_{1,i}$, $\sigma_{1,i}$, etc.) and $g_i(t)$.

3.2.2 Dynamics of the Average Production Model

In order to estimate the parameters, one needs to have expressions for m_i, $e_{Y,i}$, p_i, $e_{R,i}$, f_R and f_Y of the previous ODEs for any time t of the cell cycle. But the interdependence between $e_{Y,i}$ and f_Y on one hand and $e_{R,i}$ and f_R on the hand raises difficulties when integrating these equations. Explicit solution for the dynamics m_i, $e_{Y,i}$, p_i, $e_{R,i}$, f_R and f_Y are therefore not easy to obtain directly.

In order to have expressions for these quantities, we have chosen to some simplifications. In the next sections, the stochastic simulations show a good correspondence between their average concentration of free RNA-polymerase and ribosomes and the ones predicted here; it will therefore justify *a posteriori* the simplifications that we make in this section.

For the RNA-polymerases, we denote by $\widetilde{\mu_1} := \sum_i \mu_{1,i}/K$ the average elongation rates of transcription and the function h such as

$$h(t) := \sum_{i=1}^{K} e_{Y,i}(t) \frac{\widetilde{\mu_1}}{\mu_{1,i}}.$$

The dynamic of h is given by summing the Equations (3.4) for i from 1 to K, and by using Equation (3.6):

$$\frac{d}{dt}h(t) = f_Y(t) \cdot \widetilde{\mu_1} \left(1 + \sum_{i=1}^{K} \frac{\lambda_{1,i}}{\mu_{1,i}} g_i(t)\right) - \beta_Y \widetilde{\mu_1} - \frac{\log 2}{\tau_D} \cdot h(t). \tag{3.9}$$

The h is simply a weighted sum of the $e_{Y,i}$ allocated RNA-polymerases. We decided to consider that such weighting has little influence, and that h does not greatly differ from the uniform sum $\sum_i e_{Y,i}$, that is to say:

$$h(t) = \sum_{i=1}^{K} e_{Y,i}(t) \frac{\widetilde{\mu_1}}{\mu_{1,i}} \quad \simeq \quad \sum_{i=1}^{K} e_{Y,i}(t) = \beta_Y - f_Y(t).$$

It would be in particular true if all elongation rates $\mu_{1,i}$ are identical for all genes (i.e. if $\mu_{1,i} \equiv \widetilde{\mu_1}$ for all i).

With this simplification, from Relation (3.9), one obtains a differential equation for f_Y

$$\frac{\mathrm{d}}{\mathrm{d}t} f_Y(t) = \widetilde{\mu_1}\beta_Y \left(\frac{\log 2}{\widetilde{\mu_1}\tau_D} + 1\right) - \widetilde{\mu_1}\left(1 + \frac{\log 2}{\widetilde{\mu_1}\tau_D} + \sum_{i=1}^{K} \frac{\lambda_{1,i}}{\mu_{1,i}} g_i(t)\right) f_Y(t). \tag{3.10}$$

One can remark that the concentrations of free RNA-polymerases is on a quick timescale. Indeed, as there are of the order of 1.4×10^3 mRNAs in the cell, see [17] that last approximately 4 minutes, see [1], it gives of the order of 6 translations per second. As a consequence, one can expect that that f_Y quickly reaches its steady state during the cell cycle. This consideration will be justified *a posteriori* by the agreement with stochastic simulations.

With these considerations, we set the derivative term of Equation (3.10) to be null, hence

$$f_Y(t) = \beta_Y \frac{1 + \dfrac{\log 2}{\widetilde{\mu_1}\tau_D}}{\displaystyle\sum_{i=1}^{K} \frac{\lambda_{1,i}}{\mu_{1,i}} g_i(t) + 1 + \frac{\log 2}{\widetilde{\mu_1}\tau_D}}.$$

In the next section it will be shown that $\log 2/(\widetilde{\mu_1} \times \tau_D) \sim 10^{-3} \ll 1$, we will therefore neglect the contribution of this term. With a similar argument for free ribosomes, we get

$$f_Y(t) = \beta_Y \frac{1}{1 + \displaystyle\sum_{i=1}^{K} \frac{\lambda_{1,i}}{\mu_{1,i}} g_i(t)} \quad \text{and} \quad f_R(t) = \beta_R \frac{1}{1 + \displaystyle\sum_{i=1}^{K} \frac{\lambda_{2,i}}{\mu_{2,i}} m_i(t)}.$$

With global quantities f_Y and f_R known, we are able to give expression for gene-specific variables. For each $i \in \{1, ..., K\}$, one can integrate Equation (3.2) and find that:

$$\frac{\mathrm{d}m_i}{\mathrm{d}t}(t) = \lambda_{1,i} g_i(t) \cdot f_Y(t) - \sigma_{1,i} m_i(t) - \frac{\log 2}{\tau_D} \cdot m_i(t).$$

With the boundary conditions of Equation (3.8), it is easy to deduce that:

$$m_i(t) = \lambda_{1,i} \frac{e^{-\sigma_{1,i}t}}{2^{t/\tau_D}} \left[\int_0^t 2^{u/\tau_D} e^{\sigma_{1,i}u} g_i(u) f_Y(u)\,\mathrm{d}u + \frac{\int_0^{\tau_D} 2^{u/\tau_D} e^{\sigma_{1,i}u} g_i(u) f_Y(u)\,\mathrm{d}u}{2e^{\sigma_{1,i}\tau_D} - 1}\right]. \tag{3.11}$$

Since the quantities g_i , f_Y are known, we have an explicit solution for m_i.

Similarly for $e_{Y,i}(t)$ and $e_{R,i}(t)$,

$$e_{Y,i}(t) = \lambda_{1,i} \frac{e^{-\mu_{1,i}t}}{2^{t/\tau_D}} \left[\int_0^t 2^{u/\tau_D} e^{\mu_{1,i}u} g_i(u) f_Y(u)\,\mathrm{d}u + \frac{\int_0^{\tau_D} 2^{u/\tau_D} e^{\mu_{1,i}u} g_i(u) f_Y(u)\,\mathrm{d}u}{2e^{\mu_{1,i}\tau_D} - 1}\right],$$

$$e_{R,i}(t) = \lambda_{2,i} \frac{e^{-\mu_{2,i}t}}{2^{t/\tau_D}} \left[\int_0^t 2^{u/\tau_D} e^{\mu_{2,i}u} m_i(u) f_R(u)\,\mathrm{d}u + \frac{\int_0^{\tau_D} 2^{u/\tau_D} e^{\mu_{2,i}u} m_i(u) f_R(u)\,\mathrm{d}u}{2e^{\mu_{2,i}\tau_D} - 1}\right].$$

Consider now the type i protein concentration. By integrating the Equation (3.2), and by considering the boundary condition of Equation (3.8), one gets the relation

$$p_i(t) = \frac{\mu_{2,i}}{2^{t/\tau_D}} \int_0^{\tau_D} \left(1 + \mathbf{1}_{\{u<t\}}\right) 2^{u/\tau_D} e_{R,i}(u)\,\mathrm{d}u. \tag{3.12}$$

As in the previous models, we are interested in average concentrations over the cell cycle. Since, in the system of ODEs, we define average concentrations over the cell cycle of free RNA-polymerases and ribosomes respectively as

$$\overline{f_Y} = \frac{1}{\tau_D}\int_0^{\tau_D} \beta_Y \frac{1}{\displaystyle\sum_{i=1}^{K}\frac{\lambda_{1,i}}{\mu_{1,i}}g_i(t)+1}\,\mathrm{d}t \quad \text{and} \quad \overline{f_R} = \frac{1}{\tau_D}\int_0^{\tau_D} \beta_R \frac{1}{\displaystyle\sum_{i=1}^{K}\frac{\lambda_{2,i}}{\mu_{2,i}}m_i(t)+1}\,\mathrm{d}t. \tag{3.13}$$

We defined similarly the concentrations $\overline{m_i}$ and $\overline{p_i}$ averaged over the cell cycle. By integrating Equations (3.12) and (3.12), it follows:

$$\overline{m_i} = \frac{\lambda_{1,i}}{\sigma_{1,i}\tau_D + \log 2}\int_0^{\tau_D} g_i(u) f_Y(u)\,\mathrm{d}u \tag{3.14}$$

$$\overline{p_i} = \frac{\lambda_{2,i}\mu_{2,i}\tau_D}{\log 2\,(\mu_{2,i}\tau_D + \log 2)}\int_0^{\tau_D} m_i(u) f_R(u)\,\mathrm{d}u. \tag{3.15}$$

Now we have expressions of the average concentrations of $\overline{m_i}$, $\overline{p_i}$, $\overline{f_R}$ and $\overline{f_Y}$ for any time t in the cell cycle that will be used in the next subsection to determine the parameters.

3.3 Estimation of Parameters

The stochastic model of this section are used to describe the production of all proteins of the cell. Recall that [1] has only considered 1018 genes, out of which only 841 have their mRNA production measured. In a first step, we only take into account the 841 genes with protein and mRNA production measured and consider that it would represent the whole genome; in Section 3.6.1 we will study the case of a simulation with a complete set of genes representing a full genome of about 2000 genes.

The determination of the model parameters $\sigma_{1,i}$ of mRNA degradation of type i , of the doubling time τ_D and the time $\tau_{R,i}$ of gene replication is the same as for the previous intermediate models (see Sections 1.2 and 2.2).

We still need to determine all reaction rates for every protein type ($\lambda_{1,i}$, $\mu_{1,i}$, $\lambda_{2,i}$ and $\mu_{2,i}$ for $i \in \{1, ..., K\}$) as well as concentration parameters of RNA-polymerases, and ribosomes (respectively β_Y and β_R), the proportion between the volume and the protein mass β_P, the mass of each proteins w_i and the copy number g_i of any gene.

Reference [1] does not give the quantities of non-allocated RNA-polymerases or ribosomes. To determine the set of parameters, we fix the average concentration of free RNA-polymerases and ribosomes. Note that we can have multiple sets of parameters depending on this choice. In the simulations, we will examine several simulations with different values for average free RNA-polymerase and ribosome concentrations to see their impact on the dynamic of the model (Section 3.5).

The rates $\mu_{1,i}$, $\mu_{2,i}$ of mRNAs and protein elongation rates can be deduced from the gene length of the i-th gene. In the description of the model, we have considered that the length of the mRNA is characterized by its length; so a rate the parameter $\mu_{1,i}$ is given by the mRNA elongation speed (39 Nucl/s in [18] for slowly growing cells) divided by the length of the i-th gene. Similarly $\mu_{1,i}$ is given by the protein elongation speed (12 aa/s in [18] for slowly growing cells) divided by the number of amino-acids coded by the i-th gene divided . The mass of each protein w_i is also deduced from the length of the gene as it determines the number of amino-acids of the protein.

What remains to determine are the concentration parameters of RNA-polymerases, and ribosomes (β_Y and β_R), the proportion between the volume and the mass of proteins β_P, as well as the activities of the gene and the mRNA (respectively $\lambda_{1,i}$ and

$\lambda_{2,i}$) in each unit of production $i \in \{1, ..., K\}$. To do so, we interpret the mRNA and protein concentration of each type measured in [1] as the average concentration of each mRNA and proteins over the cell cycle of this model (respectively $\overline{m_i}$ and $\overline{p_i}$). Moreover, as previously said, the average concentrations of free RNA-polymerases $\overline{f_Y}$ and free ribosomes $\overline{f_R}$ are fixed.

We want now to compute β_P, β_Y, β_R, $\lambda_{1,i}$ and $\lambda_{2,i}$ based on known values for $\overline{f_Y}$, $\overline{f_R}$, $\overline{m_i}$ and $\overline{p_i}$. We determine the parameter β_P. In the description of the stochastic model, Equation (3.1) states that at any moment, the volume is considered to be proportional to the total mass of proteins. Interpreting $\overline{p_i}$ as the average concentration of the protein of type i leads by integration of Equation (3.1) to

$$\beta_P = \sum_{i=1}^{K} w_i \overline{p_i}.$$

We continue with the parameters relevant to the transcription: $\lambda_{1,i}$ and β_Y. With Equations (3.13) and (3.15), $\beta_Y, \lambda_{1,1}, ..., \lambda_{1,K}$ are solution of the system

$$\begin{cases} \beta_Y &= \overline{f_Y} \left(\dfrac{1}{\tau_D} \displaystyle\int_0^{\tau_D} \left(\sum_{i=1}^{K} \dfrac{\lambda_{1,i}}{\mu_{1,i}} g_i(t) + 1 \right)^{-1} \mathrm{d}t \right)^{-1} \\ \lambda_{1,i} &= \overline{m_i} \cdot (\sigma_{1,i}\tau_D + \log 2) \cdot \left(\displaystyle\int_0^{\tau_D} g_i(u) f_Y(u)\ \mathrm{d}u \right)^{-1} \quad \forall i \in \{1, ..., K\}. \end{cases} \tag{3.16}$$

Since $\overline{f_Y}$, $\overline{m_i}$ and $g_i(t)$ have already been settled, we can use a fixed point optimization procedure to determine β_Y and all $\lambda_{1,i}$. Then, as these parameters are determined, we now have an explicit expression for $f_Y(t)$ for any time t of the cell cycle.

We have to determine the parameters relevant to translation, namely $\lambda_{2,i}$ and β_R. Here again, we use a fixed point optimization procedure to deliver the result. With Equation (3.13) and the expression of $\overline{p_i}$ in Equation (3.15), $\beta_R, \lambda_{2,1}, ..., \lambda_{2,K}$ are solutions of the system

$$\begin{cases} \beta_R &= \overline{f_R} \times \left(\dfrac{1}{\tau_D} \displaystyle\int_0^{\tau_D} \left(\sum_{i=1}^{K} \dfrac{\lambda_{2,i}}{\mu_{2,i}} m_i(t) + 1 \right)^{-1} \mathrm{d}t \right)^{-1} \\ \lambda_{2,i} &= \overline{p_i} \times \left(\dfrac{\mu_{2,i}\tau_D}{\log 2\,(\mu_{2,i}\tau_D + \log 2)} \displaystyle\int_0^{\tau_D} m_i(u) f_R(u)\ \mathrm{d}u \right)^{-1} \quad \forall i \in \{1, ..., K\}. \end{cases} \tag{3.17}$$

By fixing the average amount of free RNA-polymerases and ribosomes, it is possible, through this procedure to determine parameters with the experimental measures.

3.4 Validation of the Average Production Model

The description of the average production through the system of ODE (Section 3.2.1) makes the computation of parameters of the stochastic model possible. We need to check that the deterministic description globally corresponds to the average behavior of the stochastic model; for instance, one has to validate that stochastic simulations with the parameters previously determined, are consistent with the number of mRNAs and proteins observed.

Here, we present the results of a particular simulation, whose parameters are presented in S3 Fig(A).Its average behavior will be compared with the expressions derived from the system of ODEs. The simulation presented here takes into account the 841 genes with protein and mRNA production described in [1], and we have fixed the number of free RNA-polymerases and ribosomes in order to compute the parameters.

The system of ODEs assumes volume growth is exponential with rate $\log 2/\tau_D$. In S3 Fig(A), the volume of the cell indeed seems to grow exponentially in the simulations; the growth rate corresponds to the expected a doubling time of τ_D.

For each type of gene, S3 Fig(B), shows the ratio between protein production observed in the simulations divided by protein production expected (and similarly for the mRNAs in inset). It appears that the correspondence is correct, especially for the highly expressed proteins. It is less precise for the protein less expressed but, globally, the correspondence seems good enough.

Computed from the stochastic simulations, the main S3 Fig(C) and S3 Fig(D) present the mean number of free RNA-polymerases and ribosomes as a function of cell volume. The mean of each free entity is not constant during the cell cycle. The dashed lines represent the expected value of free entities given by the model of ODEs (Equation (3.13)). It is indeed a good approximation for the behavior of free RNA-polymerases and ribosomes. The stochastic simulation displays relative quick timescale for the evolution of free RNA-polymerases (of the order of the second) and even quicker for the free ribosomes (insets of S3 Fig(C) and S3 Fig(D)).

All these results support the idea that the expressions derived from the system of ODEs are accurate to describe the average behavior of the stochastic model.

3.5 Impact of Free RNA-polymerases and Ribosomes

As in Section 3.2.1, the parameter computation supposes that the average concentrations of free RNA-polymerases $\overline{f_Y}$ and ribosomes $\overline{f_R}$ are fixed. In S4 Fig is presented several simulations where the average concentrations of these free entities are changed.

3.5.1 Few Free Ribosomes and Many Free RNA-polymerases

The first simulation, corresponding to S4 Fig(A) and S4 Fig(B), considers a low concentration of free ribosomes and a high concentration of free RNA-polymerases. This situation seems to reasonable in this biological setting. Consequently, free ribosomes and free polymerases are subject to a large competition between transcripts (see [19] in the case of the yeast). At the same time, the parameters are fixed in such a way that most of RNA-polymerases are non-allocated, i.e. not bound on the DNA, see [20], see Figure 4 of the main article and S3 Fig(A). S4 Fig(A) compares the variance of the complete stochastic model, with the one predicted by the previous model (with gene replication and random partitioning); it shows that for 90% of the genes, protein variance ratio is above 0.9 (the mean of the ratio is 0.96).

In these simulations, we look at the distributions of free RNA-polymerases and ribosomes. In S4 Fig(B), we show these distributions at three different phases in the cell cycle: we have selected cells of a given volume, either $1.40\,\mu m^3$,$1.95\,\mu m^3$ or $2.50\,\mu m^3$, which correspond to the beginning, middle and end of the cell cycle. These distributions change as the volume increase (so that the average follows the curves shown in S3 Fig(C) and S3 Fig(D)).

In order to interpret the observed distributions of free RNA-polymerases and ribosomes at a certain extend, we can propose a simplified model of RNA-polymerase and ribosome allocation for which translation and the translation are then considered separately, and that there is no notion of cell growth. The idea would be to approach the "local" steady state of RNA-polymerases and ribosomes before any significant change in the volume (for more details about the simplified model, see Section 3.8).

This simplified description predicts that for a given volume V, the distribution of free RNA-polymerases and ribosomes would be both a binomial distribution. These predicted binomial distributions are plotted in S4 Fig(B) in thick lines. In the RNA-polymerase case, the binomial distribution globally fit the histograms. The

ribosome distribution is singular: the parameters of the binomial distribution (N, ϕ) are such that $\phi \ll N$. It is due to the low concentration of free ribosomes chosen for the parameters computation. But even this denatured case shows a good correspondence between the binomial distribution and the simulation histograms.

3.5.2 Influence of Free RNA-polymerase Concentration

By keeping the low concentration of free ribosomes, we have produced a series of parameters where the average concentration of free RNA-polymerases was fixed successively to 1, 10, 100 and 1000 copies/μm^3. In each case, we have deduced a set of parameters, where the affinity constants $\lambda_{1,i}$, $\lambda_{2,i}$ are still calculated so that average mRNA and protein concentrations still correspond to the experimental measures. By performing simulations, we observe that protein variability remains in the same order of magnitude. Even more, as shown in S4 Fig(C), the gap between the multi-protein model and the previous gene centered model (with volume growth, random partition and gene replication), seems to be reduced for the lowest free RNA-polymerase concentration: the variance ratio between the two models is 0.96 (90% of the genes have a variance ratio above 0.92).

Even for extremely low RNA-polymerase concentrations, the distribution of free RNA-polymerases and ribosomes are still well predicted by the simplified model presented in Section 3.8 (see S4 Fig(D)).

3.5.3 Influence of Free Ribosome Concentration

With a high concentration of free RNA-polymerases, we observe the influence of the quantity of free ribosomes on protein variance. We have computed a set of parameters based on average concentrations of non-allocated ribosomes of 1, 10, 100 and 1000 copies/μm^3. It can be first remarked that for very high free concentrations, the binomial fit of the simplified model (described in Section 3.8) is not relevant to describe the free ribosome distribution.

In this case again, changes to the average concentration of free ribosomes are negligible. As the average concentration of free ribosomes increases, the variance of each protein decreases. As shown in S4 Fig(E), for a concentration of 1000 copies/μm^3, the variance of the multi-protein represents on average 0.98 of the one predicted by the gene-centered model (90% of the genes have a variance ratio above 0.93).

Fluctuations in the number of free ribosomes seem to be the main source of the small additional variability observed in the multi-protein model (compared with the previous intermediate model); and this effect seems less important as the number of free ribosomes is high. But in real bacteria, the number of free ribosomes usually seems quite low due to the high cost of ribosome production; then, a low number of free ribosomes (like in the simulation of Section 3.5.1) seems more plausible than this simulation.

To confirm the specific influence of fluctuations of ribosomes on protein variability, we have performed simulations with a modified version of the model. The multi-protein model has been changed in such a way that the concentration of non-allocated ribosomes is fixed during the whole simulation (meanwhile the free RNA-polymerases are still fluctuating). Results about protein variability are similar to what is shown in S4 Fig(E): the variance of each protein concentration is equivalent to what was described by the gene-centered model.

3.6 Other Possible Influences on Protein Variability

In this section, based on the set of parameters of the simulation Section 3.5.1 (with few free ribosomes and many free RNA-polymerases), we make variations on some modeling

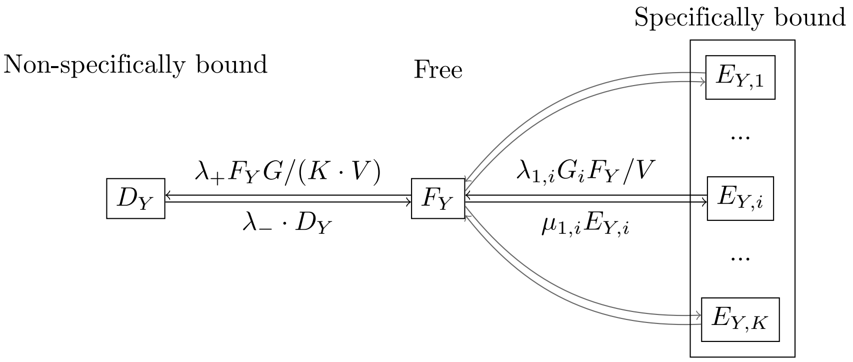

S8 Fig. Model variant with non-specific RNA-polymerase binding. An RNA-polymerase can be either among the specifically bound on the DNA (in $E_{Y,i}$ for $i \in \{1, ..., K\}$) or free (among F_Y) or non-specifically bound on the DNA (among D_Y).

choices for some cellular mechanisms: a larger set of genes, RNA-polymerases and ribosomes as a result of gene expression, the introduction of RNA-polymerase non-specific binding on the DNA, considering uncertainty in the division and DNA replication processes, etc. We will show that protein variability is quite robust to any of these changes: as for the results presented in Section 3.5.1, protein variance is still increased by at most 10% compared to the gene-centered model.

3.6.1 Additional Genes

The genome of *E. coli* has approximately 2000 expressed genes. But the measures of [1] take into account only a part of it. A total 1018 protein types were considered in this reference, and among them, only 841 types have their mRNA production estimated. In order to better represent the complete genome of the bacteria, we have created a set of parameters with an extended pool of additional randomly created genes so that the total number of genes would be 2000.

For each new gene, we have sampled its average protein and mRNA concentration, an mRNA lifetime and gene position. By studying the data of [1], we have investigated the possible statistical correlations between these quantities; it appears that only the mRNA and protein concentration are correlated (as it is shown in the Figure 7B of the main article). We therefore have sampled the mRNAs lifetime and the gene position and length independently from the two other quantities.

As in the dataset, the genes are evenly distributed in the DNA, the gene position is assumed to be uniformly distributed. The empirical mRNA lifetime distribution fitted a log-normal distribution; we have chosen the mRNA lifetime accordingly.

For the mRNA and the protein expression, we have taken into account their correlation. The first step of the procedure is to sample protein production of the new gene according protein empirical distribution of the dataset. We then obtain a realistic protein production for the new gene. In a second step, the mRNA production is chosen depending on its protein production already determined. We have subdivised the dataset in 10 classes according to protein production (see the different colors of Figure 5A of the main article); we then consider the classes in which protein production of the new gene falls in. We then sample its mRNA production according to the empirical distribution of the mRNA productions of this specific class only. Thus, the mRNA production of the new gene is sampled with a specific distribution that depends on its protein production With this procedure, the newly created genes have a protein and mRNA productions that seem to be in adequation with the original dataset (see

Figure 5A of the main article).

Simulations with the completed genome show no significant difference in terms of protein variability. In particular, the variance ratio between protein concentration of the gene-centered model and the multi-protein model is not different as in Section 3.5.1.

3.6.2 Production of RNA-polymerase and Ribosomes

In the complete stochastic model as it was presented in Section 3.1, all ribosomes and all RNA-polymerases are supposed to have constant concentrations (respectively β_R and β_Y). In reality, both RNA-polymerases and ribosomes are composed of different subunits, each subunit is either a protein or, in the case of ribosomes, a functional RNA. The variability of the production of these subunits can have an overall impact on the global production.

We have performed a preliminary simulation that takes into account this aspect: the goal is not to have a precise description of mechanisms of RNA-polymerase and ribosome productions, but rather to have an insight in the magnitude of additional variability it can induce. In this version of the model, the expression of one gene represents RNA-polymerase production and the expression of another gene represents the ribosome production. It refers to a case where the RNA-polymerases and ribosomes would be composed of only one proteic subunit.

We therefore created two genes, whose protein production was fixed to correspond to the measured concentration of RNA-polymerases and ribosomes. The mRNA production and lifetime, the gene position and length have been chosen by the same procedure as described in the previous subsection.

This simulation brings an additional variability in the growth rate: volume growth is more variable. These fluctuations are directly correlated with the number of ribosomes in the cell (Figure 5B of the main article, above). But surprisingly, these additional variability has no significant impact in protein variability. The Figure 5B of the main article (below) also shows the distribution of the protein FabH for cells of different volumes. This case does not differ from the case where the total amount of RNA-polymerases and ribosomes are in constant concentration.

We can propose a possible interpretation of these results. The fluctuations in the total number of ribosomes seems to influence primarily the speed of growth as shown in Figure 5B of the main article. When ribosomes are produced, it accelerates the global production of all types of proteins thus increasing the volume. As a consequence, both the production of each type of protein and the volume are co-regulated. Fluctuations in the total number of ribosomes affect volume growth and the production of the i-th protein in the same way such as in a cell of a given volume, the i-th protein distribution is relatively unchanged.

3.6.3 Non-specifically Bound Polymerases

In the complete stochastic model as it was described in Section 3.1, RNA-polymerases are either on the DNA involved in a transcription process, or is among the F_Y free RNA-polymerases that freely evolve in the cytoplasm. But it has been experimentally shown that large portion of RNA-polymerases can bind non-specifically on the DNA, without initiating transcription. For instance, [20] estimated that around 90% of the RNA-polymerases are non-specifically bound to the DNA.

We have created an alternative version of the stochastic model to introduce a third possible class for RNA-polymerases. RNA-polymerases can also bound non-specifically on the DNA. The binding rate is modeled as follows: at any time t, a free RNA-polymerase bind on the DNA at a rate that depends on the number of free RNA-polymerases $F_Y(t)$ and on the DNA concentration $G(t)/(K \cdot V(t))$ (where

$G(t) = \sum_i G_i(t)$); the global rate is hence $\lambda_+ F_Y(t)G(t)/(K \cdot V(t))$ where λ_+ is a parameter that represents the natural affinity of RNA-polymerases for the DNA. Once an RNA-polymerase is bound, it is released in a time represented by an exponential random variable of rate λ_- (see S8 Fig).

We performed a simulation where the parameters λ_+ and λ_- are chosen such that around 90% of the RNA-polymerases are sequestered on the DNA at any time, as it was the case in [20]. The variability of protein concentration does not seem to be impacted in this case.

3.6.4 Uncertainty in the Replication Initiation and Division Timing

In the complete stochastic model as it was initially described, replication initiation and division occur when the cell reaches the respective volumes of V_I and $2V_0$. In practice it does not occur in this way. We propose here a modification of the stochastic model to take into account this aspect.

The way replication and division occur is still a disputed topic, see for example [15, 21–23]. For the division, one hypothesis (referred as "sizer model") is that the division decision depends on the current size of the cell (the size can refer to the mass or the volume, but as explained in Section 3.1, the density constraint, see [24], ensures a proportionality relation between these two quantities). With this hypothesis, at any instant, the instantaneous probability to divide depends only on the current cell size. In a first approximation, the cell size distributions observed experimentally can be explained by this "sizer model", see [13, 23]. It is therefore this framework that we have considered to represent the cell division decision.

At time t of the simulation, with a cell of volume $V(t)$, we introduce an instantaneous division rate $b_D(V(t))$ with b_D is a positive function (the probability to divide between times t and $t + \mathrm{d}t$ is given by $b_D(V(t))\ \mathrm{d}t$). The division decision is hence only volume dependent. The function b_D is chosen so that the division occurs around the volume $2V_0$ with $V_0 = 1.3\,\mu\mathrm{m}^3$ and division precision can be fixed (for more information about the function b_D, see Chapter 4 of [7]).

Similarly for the replication initiation decision, the stochastic model initially described considers a fixed volume V_I at which the DNA replication is initiated. We introduce variability in this cell decision, in the same way as we do for the division: at time t, we consider a replication initiation rate $b_I(V(t))$ such as the function b_I is chosen in order to have a replication initiation that occurs around volume V_I.

We did several simulations with different functions b_D and b_I in order to have different precisions in the division and replication initiation decisions. Protein variability does seem to be changed significantly by any of these scenarios.

3.6.5 Deterministic Time for Replication

When the stochastic model has been initially presented (in Section 3.1), we have proposed two ways to model the time of DNA replication initiation τ_I . It can either be a deterministic time after the last division, or it can happen when the cell reaches the specific volume V_I. We have checked that this modeling choice has no significant influence on the global dynamic of the system, in particular in the protein noise.

3.7 Environmental State Decomposition

In order to perform the environmental state decomposition, one has to specify the environment Z with which the conditioning is made in Equation (8) of the main article. In order to have a decomposition analog to the result of the dual reporter technique, one has to take into account the environmental aspects that would be similar in the

expression of two identical genes in the same cell. In our model, two gene expressions in the same cell would undergo the same volume growth (as it was the case for the first two intermediate models), the same number of free RNA-polymerases, and the same number of free ribosomes. But on the contrary, the division would be specific to each expression. For a protein of type i, the decomposition would therefore be:

$$\mathrm{Var}\left[P_i/V\right] = \underbrace{\overline{\left\langle \overline{\mathrm{Var}}\left[P_i(t)/V(t)|\,(V(t),F_Y(t),F_R(t))\right]\right\rangle}}_{\overline{\mathrm{Var}}_{int}[P_i/V]} + \underbrace{\overline{\mathrm{Var}}\left[\overline{\langle P_i(t)/V(t)|\,(V(t),F_Y(t),F_R(t))\rangle}\right]}_{\overline{\mathrm{Var}}_{ext}[P_i/V]}.$$

Estimations of $\overline{\mathrm{Var}}_{int}\left[P_i/V\right]$ and $\overline{\mathrm{Var}}_{ext}\left[P_i/V\right]$ are directly made on the results of the simulations. The result is that the extrinsic contribution of the variance $\overline{\mathrm{Var}}_{ext}\left[P_i/V\right]$ represent only a very small portion of $\overline{\mathrm{Var}}\left[P_i/V\right]$ for any gene i.

3.8 Simplified Models for Transcription and Translation

Reference [25] proposes a multi-protein model for translation, with a limited number of ribosomes, where each type of mRNA is supposed to be in constant quantities and where the maximum number of ribosomes on one single mRNA is limited. The system also evolves in a fixed volume as it is the case for classic models.

In order to have a prediction for the number of respectively free RNA-polymerases and free ribosomes; we consider two analog models that are slightly simplified versions of the model of [25]. They respectively represent the transcription and translation part and they are completely independent.

The goal is, for each of these models, to provide the equivalent of the first results of [25] and we will show that the expected distribution of free RNA-polymerases (or free ribosomes) is binomial in these simplified cases.

A Model for Transcription

As explained in Section 3.5.1, one can interpret the model of [25] as taking place in a fixed volume V (it would correspond to a small portion of the cell cycle in the complete stochastic model, a portion where the volume of the cell does not change much). We also consider that the gene copy of each unit of production remains constant; as a consequence, the gene copy number of the i-th gene G_i is constant and known. As in the complete stochastic model, and contrary to the model of [25], we consider that there is no limiting number of elongating RNA-polymerases on one gene.

In a pool of K genes, denote by N_Y the constant total number of polymerases. We consider the random variables $E_{Y,i}$ for $i \in \{1,...,K\}$ be the number of RNA-polymerases attached to the i-th gene. As a consequence, the random variable

$$F_Y := N_Y - \sum_{i=1}^{K} E_{Y,i} \tag{3.18}$$

is the number of free RNA-polymerases in the system.

The process $X(t) = (E_{Y,i}(t), i \in \{1,...K\})$ takes place in the state place t the subset S of $\mathbb{N}^K$ such as

$$S := \left\{ x \in \mathbb{N}^K, \sum_{i=1}^{K} x_i \leq N_Y \right\}.$$

There are at most N_Y RNA-polymerases that can be attached to genes at the same time. We can describe the Markov process transition by the following Q-matrix: by setting the

vector $e_i = (\delta_{i'=i})_{i' \in \{1,\dots,K\}}$ (δ is used here as the Kronecker delta), for any $x, y \in S$,

$$\begin{cases} q(x, x+e_i) = \lambda_{1,i} G_i \lambda_{1,i} f(x)/V & \text{for any } i \in \{1, ...K\}, \\ q(x, x-e_i) = \mu_{1,i} x_i & \text{for any } i \in \{1, ...K\}, \text{ if } x_i > 0, \\ q(x,y) = 0 & \text{if } \|x-y\| > 1. \end{cases}$$

where

$$f_Y(x) := N_Y - \sum_{i=1}^{K} x_i$$

the number of free RNA-polymerases. Equation (3.18) leads in particular to $f(x - e_i) = f(x) + 1$ for all i.

As in [25], we are looking for an invariant reversible probability measure π of this Markov process, i.e. for any $i \in \{1, \dots, K\}$,

$$\pi(x)\mu_{1,i} x_i = \pi(x - e_i) \cdot \lambda_{1,i} G_i \left(f_Y(x) + 1\right)/V.$$

Proposition 15. *The invariant measure π of the number of RNA-polymerases in each gene has the following form*

$$\pi(x) = \frac{1}{Z} \cdot \frac{1}{f_Y(x)!} \prod_{i=1}^{K} \frac{(G_i \lambda_{1,i}/(V\mu_{1,i}))^{x_i}}{x_i!}$$

for any $x \in S$ and with $Z > 0$ the normalization constant.

Proof. We only need to check that π satisfies Equation (3.8). For a gene $i \in \{1, ..., K\}$, we take $x \in S$ such that $x - e_i \in S$.

$$\begin{aligned} \pi(x)\mu_{1,i}x_i &= \frac{1}{Z} \cdot \frac{1}{f_Y(x)!} \prod_{i'=1}^{K} \frac{(G_{i'}\lambda_{1,i'}/(V\mu_{1,i'}))^{x_{i'}}}{x_{i'}!} \mu_{1,i} x_i \\ &= \frac{1}{Z} \cdot \frac{1}{(f_Y(x)+1)!} \prod_{i' \neq i}^{K} \left(\frac{(G_{i'}\lambda_{1,i'}/(V\mu_{1,i'}))^{x_{i'}}}{x_{i'}!} \right) \cdot \frac{(G_i\lambda_{1,i}/(V\mu_{1,i}))^{(x_i - 1)}}{(x_i - 1)!} \cdot \\ &\qquad \frac{G_i \lambda_{1,i}}{V} (f_Y(x) + 1) \\ &= \pi(x - e_i) \cdot G_i \lambda_{1,i} (f_Y(x) + 1)/V. \end{aligned}$$

So π satisfies Equation (3.8). □

We can now derive from the previous proposition the steady state distribution of F_Y, the number of free RNA-polymerases of the process.

Proposition 16. *The distribution of the number of free polymerases F_Y is given by*

$$\mathbb{P}\left[F_Y = n\right] = \binom{N_Y}{n} \frac{\Lambda_Y^{N_Y - n}}{(1 + \Lambda_Y)^{N_Y}},$$

with Λ defined such as

$$\Lambda_Y := \sum_{i=1}^{K} G_i \lambda_{1,i}/(V\mu_{1,i}).$$

F_Y follows a binomial distribution $\mathcal{B}(\phi, N)$ with parameters $\phi = (1 + \Lambda_Y)^{-1}$ and $N = N_Y$.

Proof. From Equation (3.8), it follows that for $n \in \{1, ..., N_Y\}$,

$$\begin{aligned}\mathbb{P}[F_Y = n] &= \sum_{x\in S} \frac{1}{Z} \cdot \frac{1}{n!} \prod_{i=1}^{K} \frac{(G_i\lambda_{1,i}/(V\mu_{1,i}))^{x_i}}{x_i!} \mathbf{1}_{\{\sum_i x_i = N_Y - n\}} \\ &= \frac{1}{Z} \cdot \frac{1}{n!} \sum_{x\in S} \prod_{i=1}^{K} \frac{(G_i\lambda_{1,i}/(V\mu_{1,i}))^{x_i}}{x_i!} \mathbf{1}_{\{\sum_i x_i = N_Y - n\}} \\ &= \frac{1}{Z_{F_Y}} \cdot \frac{1}{n!} \mathbb{P}\left[\sum_{i=1}^{K}\sum_{k=1}^{G_i} C_{i,k} = N_Y - n\right]\end{aligned}$$

with $\forall i \in \{1, \ldots, K\}$ and $\forall k \in \{1, \ldots, G_i\}$, $C_{i,k} \sim \mathcal{P}(\lambda_{1,i}/(V\mu_{1,i}))$.

Since the independent random variables $C_{p,k}$ are Poisson, their sum is also Poisson with parameter $\Lambda := \sum_{i=1}^{K} G_i\lambda_{1,i}/(V\mu_{1,i})$. By summing up the previous relation, one gets that

$$\begin{aligned}1 &= \frac{1}{Z_{F_Y}} \cdot \sum_{n=0}^{N_Y} \frac{1}{n!} \mathbb{P}[C_{1,1} = N_Y - n] \\ &= \frac{1}{Z_{F_Y}} \cdot \sum_{n=0}^{N_Y} \frac{1}{n!} e^{-\Lambda} \frac{\Lambda^{N_Y-n}}{(N_Y-n)!} \qquad = \frac{1}{Z_{F_Y}} \cdot \frac{1}{N_Y!} e^{-\Lambda} \cdot \sum_{n=0}^{N_Y} \binom{N_Y}{n} \Lambda^{N_Y-n},\end{aligned}$$

hence $Z_{F_Y} = e^{-\Lambda}(1+\Lambda)^{N_Y}/N_Y!$. □

A Model for Translation

The model for translation considered here is analogous to the transcription case. We still consider that the volume V is fixed and that for each gene, the number M_i of mRNAs of type i is known and constant (because of these, the process describes here is independent of transcription). As in the complete stochastic model, and contrary to the model of [25], we consider that there is no limiting number of elongating ribosomes on one mRNA.

Similarly to the transcription, we can define N_R (the total number of ribosomes), $E_{R,i}$ (the number of ribosomes elongating an mRNA of type i) and F_R (the number of free ribosomes) such as

$$F_R := N_R - \sum_{i=1}^{K} E_{R,i}.$$

The rate at which a ribosome is sequestered on a type i mRNA is therefore $M_i\lambda_{2,i}/V$, and the rate at which an elongation terminates on a type i mRNA is $\mu_{2,i}E_{R,i}$.

As this model is analog to the transcription case, we can also prove that

Proposition 17. *The number of free ribosomes F_R follows*

$$\mathbb{P}[F_R = n] = \binom{N_R}{n} \frac{\Lambda_R^{N_R-n}}{(1+\Lambda_R)^{N_R}},$$

with Λ_R defined such as

$$\Lambda_R := \sum_{i=1}^{K} M_i\lambda_{2,i}/(V\mu_{2,i}),$$

F_R follows a binomial distribution $\mathcal{B}(\phi, N)$ for which $\phi = (1+\Lambda_R)^{-1}$ and $N = N_R$.

References

1. Taniguchi Y, Choi PJ, Li GW, Chen H, Babu M, Hearn J, et al. Quantifying *E. coli* proteome and transcriptome with single-molecule sensitivity in single cells. Science. 2010;329(5991):533–538. doi:10.1126/science.1188308.

2. Robert P. Stochastic networks and queues. Berlin; New York: Springer; 2010.

3. Kingman JFC. Poisson processes. No. 3 in Oxford Studies in Probability. Oxford: Oxford University Press; 1993.

4. Gillespie DT. Exact stochastic simulation of coupled chemical reactions. The Journal of Physical Chemistry. 1977;81(25):2340–2361. doi:10.1021/j100540a008.

5. Elowitz MB, Levine AJ, Siggia ED, Swain PS. Stochastic gene expression in a single cell. Science. 2002;297(5584):1183–1186. doi:10.1126/science.1070919.

6. Hilfinger A, Paulsson J. Separating intrinsic from extrinsic fluctuations in dynamic biological systems. Proceedings of the National Academy of Sciences of the United States of America. 2011;108(29):12167–12172. doi:10.1073/pnas.1018832108.

7. Dessalles R. Stochastic models for protein production: the impact of autoregulation, cell cycle and protein production interactions on gene expression. Université Paris-Saclay. Paris; 2017.

8. Wallden M, Fange D, Ullman G, Marklund EG, Elf J. Fluctuations in growth rates determine the generation time and size distributions of *E. coli* cells. arXiv:150403145 [q-bio]. 2015;.

9. Grant MA, Saggioro C, Ferrari U, Bassetti B, Sclavi B, Lagomarsino MC. Dnaa and the timing of chromosome replication in es-cherichia coli as a function of growth rate. BMC Systems Biology. 2011;5(1):201. doi:10.1186/1752-0509-5-201.

10. Zhou J, Rudd KE. Ecogene 3.0. Nucleic Acids Research. 2013;41(Database issue):D613–624. doi:10.1093/nar/gks1235.

11. Collins JF, Richmond MH. Rate of growth of *Bacillus cereus* between divisions. Journal of General Microbiology. 1962;28(1):15–33. doi:10.1099/00221287-28-1-15.

12. Sharpe ME, Hauser PM, Sharpe RG, Errington J. *Bacillus subtilis* cell cycle as studied by fluorescence microscopy: constancy of cell length at initiation of dna replication and evidence for active nucleoid partitioning. Journal of Bacteriology. 1998;180(3):547–555.

13. Robert L, Hoffmann M, Krell N, Aymerich S, Robert J, Doumic M. Division in *Escherichia coli* is triggered by a size-sensing rather than a timing mechanism. BMC Biology. 2014;12(1):17. doi:10.1186/1741-7007-12-17.

14. Goelzer A, Fromion V, Scorletti G. Cell design in bacteria as a convex optimization problem. Automatica. 2011;47(6):1210–1218. doi:10.1016/j.automatica.2011.02.038.

15. Wang P, Robert L, Pelletier J, Dang WL, Taddei F, Wright A, et al. Robust growth of *Escherichia coli*. Current biology: CB. 2010;20(12):1099–1103. doi:10.1016/j.cub.2010.04.045.

16. Borkowski O, Goelzer A, Schaffer M, Calabre M, Mäder U, Aymerich S, et al. Translation elicits a growth rate-dependent, genome-wide, differential protein production in *Bacillus subtilis*. Molecular Systems Biology. 2016;12(5):870. doi:10.15252/msb.20156608.

17. Neidhardt FC, Umbarger HE. Chemical composition of *Escherichia coli*. In: *Escherichia coli* and *Salmonella*: cellular and molecular biology. 2nd ed. ASM Press; 1996.

18. Bremer H, Dennis PP. Modulation of chemical composition and other parameters of the cell at different exponential growth rates. In: *Escherichia coli* and *Salmonella*: cellular and molecular biology. 2nd ed. ASM Press; 1996.

19. Warner JR, Vilardell J, Sohn JH. Economics of ribosome biosynthesis. Cold Spring Harbor Symposia on Quantitative Biology. 2001;66:567–574. doi:10.1101/sqb.2001.66.567.

20. Klumpp S, Hwa T. Growth-rate-dependent partitioning of rna polymerases in bacteria. Proceedings of the National Academy of Sciences. 2008;105(51):20245–20250. doi:10.1073/pnas.0804953105.

21. Tyson JJ, Diekmann O. Sloppy size control of the cell division cycle. Journal of Theoretical Biology. 1986;118(4):405–426. doi:10.1016/S0022-5193(86)80162-X.

22. Soifer I, Robert L, Barkai N, Amir A. Single-cell analysis of growth in budding yeast and bacteria reveals a common size regulation strategy. arXiv:14104771 [cond-mat, q-bio]. 2014;.

23. Osella M, Nugent E, Lagomarsino MC. Concerted control of *Escherichia coli* cell division. Proceedings of the National Academy of Sciences. 2014;111(9):3431–3435. doi:10.1073/pnas.1313715111.

24. Marr AG. Growth rate of *Escherichia coli*. Microbiological Reviews. 1991;55(2):316–333.

25. Fromion V, Leoncini E, Robert P. A stochastic model of the production of multiple proteins in cells. SIAM Journal on Applied Mathematics. 2015;75(6):2562–2580. doi:10.1137/140994782.
